# The CREG1-FBXO27-LAMP2 axis alleviates diabetic cardiomyopathy by promoting autophagy in cardiomyocytes

**DOI:** 10.1038/s12276-023-01081-2

**Published:** 2023-09-01

**Authors:** Dan Liu, Ruinan Xing, Quanyu Zhang, Xiaoxiang Tian, Yanping Qi, Haixu Song, Yanxia Liu, Haibo Yu, Xiaolin Zhang, Quanmin Jing, Chenghui Yan, Yaling Han

**Affiliations:** State Key Laboratory of Frigid Zone Cardiovascular Diseases, Cardiovascular Research Institute and Department of Cardiology, General Hospital of Northern Theater Command, Shenyang, China

**Keywords:** Macroautophagy, Heart failure

## Abstract

Autophagy plays an important role in the development of diabetic cardiomyopathy. Cellular repressor of E1A-stimulated genes 1 (CREG1) is an important myocardial protective factor. The aim of this study was to investigate the effects and mechanisms of CREG1 in diabetic cardiomyopathy. Male C57BL/6 J mice, *Creg1* transgenic mice and cardiac-specific knockout mice were used to establish a type 2 diabetes model. Small animal ultrasound, Masson’s staining and western blotting were used to evaluate cardiac function, myocardial fibrosis and autophagy. Neonatal mouse cardiomyocytes (NMCMs) were stimulated with palmitate, and the effects of CREG1 on NMCMs autophagy were examined. CREG1 deficiency exacerbated cardiac dysfunction, cardiac hypertrophy and fibrosis in mice with diabetic cardiomyopathy, which was accompanied by exacerbated autophagy dysfunction. CREG1 overexpression improved cardiac function and ameliorated cardiac hypertrophy and fibrosis in diabetic cardiomyopathy by improving autophagy. CREG1 protein expression was decreased in palmitate-induced NMCMs. CREG1 knockdown exacerbated cardiomyocyte hypertrophy and inhibited autophagy. CREG1 overexpression inhibited cardiomyocyte hypertrophy and improved autophagy. LAMP2 overexpression reversed the effect of CREG1 knockdown on palmitate-induced inhibition of cardiomyocyte autophagy. CREG1 inhibited LAMP2 protein degradation by inhibiting the protein expression of F-box protein 27 (FBXO27). Our findings indicate new roles of CREG1 in the development of diabetic cardiomyopathy.

## CREG1: A Potential Solution for Cardiac Dysfunction & Fibrosis

Diabetic cardiomyopathy, a fatal complication of diabetes, can be alleviated by a protein called CREG1, according to a new study. Researchers found that CREG1 expression was reduced in the hearts of diabetic mice, and that increasing CREG1 levels improved heart function and reduced fibrosis. The protein works by promoting autophagy, a process that helps maintain cell health by breaking down damaged components. By boosting autophagy in heart cells, CREG1 could help prevent the development of diabetic cardiomyopathy. This discovery could pave the way for new therapeutic strategies to treat this debilitating condition.

## Introduction

Diabetes is a chronic disease that is prevalent worldwide, and type 2 diabetes is the most common type. Cardiovascular diseases are the leading cause of death in patients with type 2 diabetes^1,2^. Diabetic cardiomyopathy is a fatal and chronic complication of diabetes in the absence of coronary artery disease, arterial hypertension, and valvular disease. Diabetic cardiomyopathy is characterized by myocardial fibrosis leading to ventricular dilation and hypertrophy, which contributes to diastolic and systolic dysfunction and eventually results in heart failure^3–5^.

The main mechanisms of diabetic cardiomyopathy in type 2 diabetes include insulin resistance, cardiomyocyte apoptosis, oxidative stress, autophagy dysfunction, and inflammation^6–9^. In particular, autophagy dysfunction plays an important role in the development of diabetic cardiomyopathy^10–12^. Autophagy is a highly conserved process of lysosome-mediated organelle and protein degradation that maintains cellular homeostasis and metabolism. Autophagy is characterized by the formation of phagophores, which engulf cytoplasmic components destined for turnover and mature into double-membrane vesicles called autophagosomes, which fuse with lysosomes to form autolysosomes, allowing for cargo degradation^13,14^. During the development of diabetic cardiomyopathy in type 2 diabetes, cardiomyocyte autophagy is inhibited, damaged organelles cannot be degraded, and harmful metabolites accumulate in cardiomyocytes, resulting in myocardial hypertrophy, myocardial fibrosis, and cardiac dysfunction^15,16^. Therefore, identifying the key molecules that regulate cardiomyocyte autophagy after diabetic cardiomyopathy could provide new strategies for the prevention and treatment of diabetic cardiomyopathy.

Cellular repressor of E1A-stimulated genes 1 (CREG1) is a small glycosylated protein that is widely expressed in a variety of cells and tissues^17–20^. Studies have revealed that CREG1 is highly expressed in the heart and plays important roles in myocardial protection^21–24^. Recombinant CREG1 protein inhibits myocardial hypertrophy and fibrosis caused by angiotensin II by promoting myocardial autophagy^25^. Recombinant CREG1 protein protects against myocardial ischemia-reperfusion injury by improving myocardial autophagy and inhibiting apoptosis^21^ and ameliorates myocardial fibrosis after myocardial infarction by inhibiting the phenotypic switching of cardiac fibroblasts^22^. Tai et al. reported that CREG1 improved cardiac function by regulating cardiomyocyte autophagy in rats with diabetic myocardial infarction^24^. However, whether CREG1 can ameliorate cardiac fibrosis and improve cardiac function in diabetic cardiomyopathy remains unknown.

In this study, we aimed to investigate the role of CREG1 in the development of diabetic cardiomyopathy and to clarify the underlying molecular mechanisms.

## Material and methods

### Animals

Eight-week-old male C57BL/6J mice, *Creg1* transgenic mice (*Creg1*-TG), *Creg1* cardiac knockout mice (*Creg1*-CKO), and their littermate controls were purchased from GemPharmatech Co., Ltd. (China). Briefly, to construct *Creg1*-CKO mice, mice bearing *Creg1*-floxed alleles (*Creg1*^fl/fl^) were crossed with transgenic mice expressing *Cre* recombinase under the cardiac muscle-specific α-MHC promoter^22^. In addition, *Creg1* transgenic mice (*Creg1*-TG) were crossed with C57BL/6 J mice. Genomic DNA isolated from the tails of *Creg1*-CKO and *Creg1*^fl/fl^ mice, *Creg1*-TG and wild type (WT) was used to genotype the animals by PCR. The primers used to identify *Creg1*-TG mice were listed in Supplementary Table 1. All male mice used had a C57BL/6 J background and were housed in a pathogen-free animal facility with an ambient temperature of 23 °C ± 2 °C and a dark-light cycle of 12-12 h.

### Experimental protocol

Eight-week-old male C57BL/6J, *Creg1*-TG, *Creg1*-CKO mice and their littermate controls were divided into two groups: the control and diabetic model (DM)^26,27^ groups. The DM group was fed a high-fat diet (HFD; fat provided 60% of total calories; Research Diet, USA). After 4 weeks of HFD feeding, the mice were intraperitoneally injected with streptozotocin (STZ, 20 mg/kg/d) for 5 days, and the cumulative dose of STZ was 100 mg/kg. The control group was fed a normal diet (ND, fat provided 10% of total calories; Research Diet) and was intraperitoneally injected with an equal amount of citrate buffer for 5 days. The criterion for the successful generation of DM was fasting blood glucose >16.7 mmol/L. C57BL/6 J mice (*n* = 6 for the control group and *n* = 8 for the DM group), *Creg1*-TG and *Creg1*^fl/fl^ mice (*n* = 7 for each group), *Creg1*-CKO mice and WT mice (*n* = 6 for the control group and *n* = 8 for the DM group) were initially included in the experiment. Mice without elevated fasting blood glucose after STZ injection were excluded from the experiment. The mice were sacrificed after 24 weeks of HFD feeding. Animal care was approved by the Ethics Committee on the Care and Use of Laboratory Animals of the General Hospital of the Northern Theater Command.

### Determination of cardiac function

Cardiac function was evaluated using small animal ultrasound (Canada) at different time points during the development of DM (0, 12, and 24 weeks). The mice were sedated by 2% isoflurane inhalation. Cardiac dimensions and function were evaluated via M-mode echocardiography using an echocardiography system with an Ms-400 linear transducer^21^. The left ventricular end-diastolic diameter and end-systolic diameter were measured on the parasternal left ventricular long axis view. The mitral valve blood flow E/A ratio (E/A), ejection fraction (EF%), and fractional shortening (FS%) were calculated using computer algorithms. All measurements were performed in a blinded manner.

### Hematoxylin & eosin (HE) staining and Masson’s trichrome staining

Heart sections were dehydrated, embedded in paraffin, cut into 3-μm-thick sections using a microtome, and mounted on slides. HE staining was performed according to routine protocols^25^. The stained sections were scanned using a microscope at 1× magnification (Zeiss, Germany). Masson’s trichrome staining was performed on paraffin sections of heart tissues to evaluate myocardial fibrosis. The stained sections were scanned using a microscope at 40× magnification (Zeiss).

### Cell culture and cell transfection

Neonatal mouse cardiomyocytes (NMCMs) were isolated from 1- to 3-day-old C57BL/6J mice using a primary cardiomyocyte isolation kit (Thermo Fisher Scientific, USA). NMCMs were cultured in Dulbecco’s modified Eagle’s medium (DMEM) supplemented with 20% fetal bovine serum (FBS) and 1% penicillin/streptomycin. The cells were plated in six-well plates and cultured at 37 °C in a 5% CO_2_ incubator. We constructed a cell model of lipotoxicity by culturing NMCMs in medium containing palmitate (PA, Sigma Aldrich, USA). PA was dissolved in bovine serum albumin (BSA), and PA was added to NMCMs growth medium for 24 h at a final concentration of 400 μM. PA-free BSA was used as a control.

To establish CREG1 knockdown cells, *Creg1* small interfering RNA and its control (si-*Creg1 and* si-control; RIBOBIO, China) were transfected into NMCMs using Lipofectamine™ RNAiMAX transfection reagent (Thermo Fisher Scientific). The target sequence for si-*Creg1* was GCCACTATCTCCACAATAA. To establish CREG1-overexpressing cells, *Creg1* and control adenoviruses (adCREG1 and adcon, the adenovirus titer for adCREG1 was 5.53 × 10^10^ PFU/ml, and the adenovirus titer for adcon was 1.58 × 10^11^ PFU/ml, OBIO Technology, China) were added to the culture medium and incubated for 24 h. Accordingly, si-*Creg1* or adCREG1 was administered to cells in full growth medium to examine the effect of CREG1 on cardiomyocyte autophagy during PA stimulation. At 24 h after adenovirus transduction or siRNA transfection, the efficiency of gene overexpression and knockdown was assessed by western blotting and real-time PCR.

CREG1-knockdown or CREG1-overexpressing NMCMs were starved in serum-free medium for 12 h and then pretreated with or without the autophagy agonist resveratrol (Res, 25 μM; Sigma Aldrich), autophagy inhibitor chloroquine (CQ, 20 μM; Sigma Aldrich) and bafilomycin A1 (Baf A1, 200 nM; MCE, USA) for 24 h.

For the rescue experiments, lysosomal-associated membrane protein 2 (LAMP2)-overexpressing adenovirus and its control (adLAMP2 and adcon, the adenovirus titer for adLAMP2 was 1.58 × 10^11^ PFU/ml, and the adenovirus titer for adcon was 1.58 × 10^11^ PFU/ml, OBIO Technology) were added to cells in the si-*Creg1* group, followed by PA stimulation for an additional 24 h. To establish F-box protein 6 (FBXO6)- or F-box protein 27 (FBXO27)-overexpressing cells, *Fbxo6* or *Fbxo27* cDNA sequences were inserted into the pcDNA3.1(+) plasmid, and the stop codon was replaced with a 3× flag tag (GENEWIZ, China). FBXO6- or FBXO27-overexpressing plasmids were transfected into NMCMs or H9C2 cells using Lipofectamine 2000 (Thermo Fisher Scientific). Three independent experiments were conducted.

### Wheat germ agglutinin staining (WGA) and F-actin staining

To analyze the cross-sectional area of cardiomyocytes, heart tissues were stained with WGA (Sigma Aldrich) according to the manufacturer’s instructions^28^. To analyze the size of cardiomyocytes in vitro, NMCMs were stained with F-actin (Sigma Aldrich) for 30 min and washed with PBS, and the nuclei were stained with DAPI. The stained sections were scanned using a microscope at 40× magnification (Zeiss).

### Lipid analysis

The levels of lipids, including triglyceride, total cholesterol, non-esterified fatty acid (NEFA), low-density lipoprotein-cholesterol (LDL-C), and high-density lipoprotein-cholesterol (HDL-C), in the myocardium of DM mice and cardiomyocytes stimulated with PA were determined by spectrophotometric methods using commercial assay kits (Nanjing Jiancheng Biotech, China)^29^.

### RNA extraction and real-time PCR

Total RNA was extracted from cells or tissues using the TRIzol method (Thermo Fisher Scientific). The mRNA expression of *Creg1, Lamp2, Fbxo6*, and *Fbxo27* in the myocardium or cardiomyocytes was measured by real-time PCR. *Gapdh* was used as the loading control. The primers listed in Supplementary Table 2 were purchased from Sangong Biotech (China). Amplification was performed as follows: 95 °C for 30 s, 30 cycles at 95 °C for 5 s and 60 °C for 30 s, and a dissolution curve from 65 °C to 95 °C (+0.5 °C one cycle). The levels of relative gene expression were calculated using the 2^−ΔΔCq^ method. The data presented were the average of at least 3 independent experiments.

### Western blotting

Total proteins were isolated from heart tissues or cells with RIPA lysis buffer (Thermo Fisher Scientific). The proteins (30 μg) were separated by SDS‒PAGE (Bio-Rad) and transferred to PVDF membranes, which were blocked with 5% skim milk. Then, the membranes were incubated at 4 °C overnight with the corresponding primary antibodies and HRP-conjugated secondary antibodies. The primary antibodies used were CREG1 (Abcam, ab191909), LAMP2 (Abcam, ab13524), LC3B (Cell Signaling Technology, USA, 3868 S), P62 (Abcam, ab109012), beclin 1 (Cell Signaling Technology, 3495 S), cathepsin D (Abcam, ab75852), FBXO6 (Bioss, China, bs-6415R), and FBXO27 (Bioss, bs-9089R). GAPDH (Cell Signaling Technology, 2118 S) was used as the internal reference.

### Immunoprecipitation (IP) and mass spectrometry

Total protein was collected from FBXO6- or FBXO27-overexpressing H9C2 cells using Pierce^TM^ IP lysis buffer (Thermo Fisher Scientific). The protein (1000 μg) was incubated with IgG (Abcam) or flag (Sigma Aldrich) antibodies and protein A/G beads (Thermo Fisher Scientific) overnight at 4 °C. The beads were washed three times and resuspended in 2× loading buffer. The expression of CREG1 and LAMP2 in the IP supernatant was examined by western blotting. The experiments were performed in triplicate.

Total protein was collected from adCREG1-expressing 3T3 fibroblasts using Pierce^TM^ IP lysis buffer (Thermo Fisher Scientific). The cell lysate (1000 μg) was incubated with IgG (Abcam) or flag (Sigma Aldrich) antibodies and protein A/G beads (Thermo Fisher Scientific) overnight at 4 °C. The beads were washed three times and resuspended in 2× loading buffer. The suspension was subjected to mass spectrometry (BioMiao Biological Technology Co., Ltd, China) to screen for proteins that might interact with CREG1.

### Immunofluorescence staining

NMCMs were plated on glass coverslips overnight, and cells treated with or without PA were fixed with 4% paraformaldehyde for 15 min and blocked with goat serum for 30 min. Then, NMCMs were incubated with specific primary antibodies against CREG1 (Sigma Aldrich) and LAMP2 (Abcam) overnight at 4 °C, followed by incubation with secondary antibodies (Thermo Fisher Scientific) for 2 h at room temperature. Cell nuclei were stained with DAPI for 5 min. Images were captured using a fluorescence microscope at 40× magnification (Zeiss).

### Measurement of fluorescent LC3 puncta

NMCMs were transfected with adCREG1, adLAMP2, si-*Creg1*, si-*Lamp2*, FBXO27 -overexpressing plasmids or corresponding controls for 24 h, transduced with an autophagy double-labeled adenovirus (mRFP-GFP-LC3; Hanbio Biotechnology, China, HB-AP210001), and stimulated with PA for an additional 24 h^30^. The cells were harvested, fixed with 4% paraformaldehyde, and then observed using a fluorescence microscope at 40× magnification (Zeiss). The double-labeled adenovirus could distinguish autophagosomes (GFP- and mRFP-positive LC3 puncta, which were yellow) and autolysosomes (GFP-negative and mRFP-positive LC3 puncta, which were red).

### Lysosome labeling with LysoTracker Red DND-99

NMCMs were differentially stimulated and stained with LysoTracker Red DND-99 (300 nM, Thermo Fisher Scientific, L-7582) for 10 min according to the manufacturer’s protocol. The excess LysoTracker was removed with culture medium. The cells were fixed and permeabilized. Coverslips were mounted using ProLong Gold antifade reagent (Thermo Fisher Scientific). Images were captured using a fluorescence microscope at 40× magnification (Zeiss) and analyzed using Image-Pro Plus v6.0 software.

### Statistical analysis

Data analysis was performed using SPSS v22.0 (IBM Inc., Armonk, NY, USA). The data were shown as the mean ± standard error of the mean (SEM). Differences between experimental and control groups were calculated using unpaired Student’s *t* tests. Differences among three or more groups were compared using one-way analysis of variance (ANOVA). Statistical significance was set at *p* < 0.05.

## Results

### CREG1 protein levels were decreased in the myocardium of type 2 diabetic mice

Compared with that in the control group, body weight in the DM group was increased after 4 weeks, while the level of fasting blood glucose was increased after 8 weeks (*p* < 0.01, Supplementary Fig. 1a, b). In addition, abnormal glucose and insulin tolerance in the DM group was observed at 24 weeks (Supplementary Fig. 1c, d). Compared with the control group, the DM group exhibited decreased diastolic and systolic functions at 24 weeks (E/A: 1.45 ± 0.14 vs. 2.17 ± 0.39; EF%: 38.87% ± 2.87% vs. 53.5% ± 1.60%; FS%: 18.56% ± 1.63% vs. 27.22% ± 1.04%, *p* < 0.01, Supplementary Fig. 1e–g). The ratio of heart weight (HW) to tibial length (TL) was used to evaluate myocardial hypertrophy. The HW/TL ratio was increased in the DM group (Supplementary Fig. 1h). HE and Masson’s trichrome staining revealed that myocardial hypertrophy and fibrosis occurred in the diabetic cardiomyopathy group at 24 weeks (Supplementary Fig. 1i, j). These results indicated that DM was successfully established.

To investigate the association between cardiac dysfunction and CREG1 expression, we evaluated the mRNA and protein expression of CREG1 in the myocardium of C57BL/6J mice at different time points. The protein expression of CREG1 was significantly decreased at 12 weeks, and the degree of the decrease was most apparent at 24 weeks (*p* < 0.01, Supplementary Fig. 1k, l); however, *Creg1* mRNA levels were unaltered (*p* > 0.05, Supplementary Fig. 1m). These results confirmed that the decrease in CREG1 expression occurred earlier than cardiac dysfunction in DM.

### CREG1 deficiency exacerbated cardiac dysfunction in type 2 diabetic mice

To clarify the role of CREG1 in the development of diabetic cardiomyopathy, *Creg1*-CKO and *Creg1*^fl/fl^ mice were used in this study. Body weight and fasting blood glucose levels in *Creg1*-CKO and *Creg1*^fl/fl^ mice in the DM group were increased compared with those in the control group (*p* < 0.01, Supplementary Fig. 2a, b). However, no difference was observed in body weight and the fasting blood glucose levels between *Creg1*-CKO and *Creg1*^fl/fl^ mice in the DM group (*p* > 0.05, Supplementary Fig. 2a, b). The HW/TL ratio was significantly higher in the DM group of *Creg1*^fl/fl^ mice than in the control group. The HW/TL ratio in the *Creg1*-CKO DM group was higher than that in the of *Creg1*^fl/fl^ DM group (*p* < 0.01, Supplementary Fig. 2c).

**Fig. 1:**
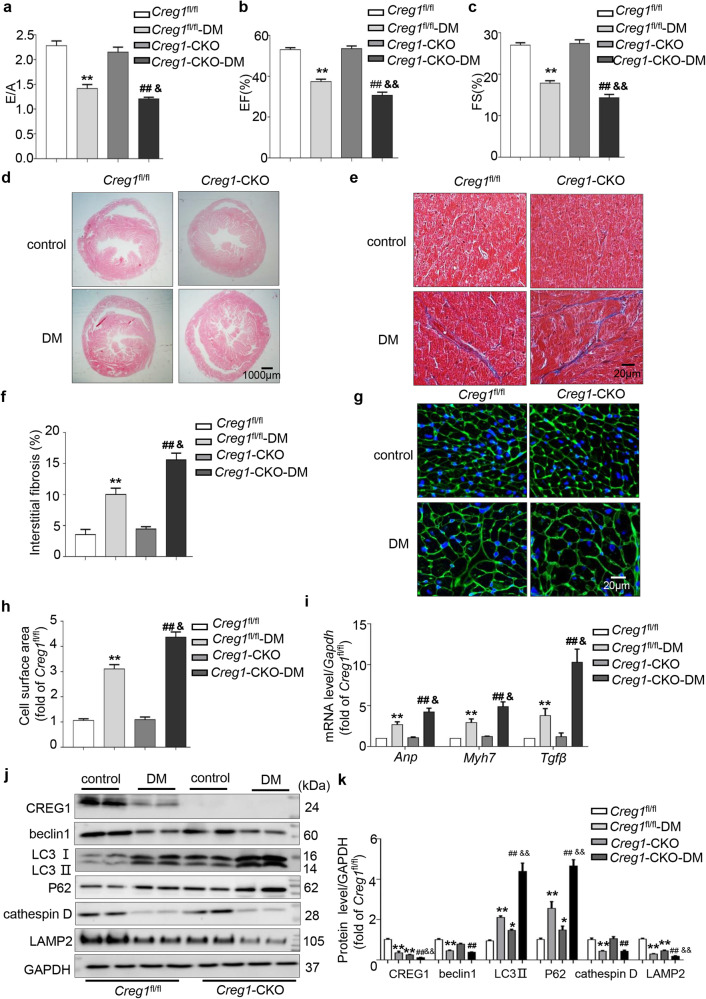
CREG1 deficiency exacerbates cardiac hypertrophy and autophagy dysfunction in type 2 diabetic mice. **a** The E/A ratio of *Creg1*^fl/fl^ and *Creg1*-CKO mice after type 2 DM was established (24 weeks, *n* = 6). **b**, **c** EF% and FS% in *Creg1*^fl/fl^ and *Creg1*-CKO mice after type 2 DM was established (24 weeks, *n* = 6). **d**–**f** HE staining and Masson’s trichrome staining of control and DM mice in the 24th week (*n* = 3). **g**, **h** WGA staining of the myocardium of control and DM mice in the 24th week (*n* = 4). **i** Effects of CREG1 deficiency on the mRNA levels of hypertrophic (*Anp* and *Myh7*) and fibrotic (*Tgfβ*) markers in the myocardium of control and DM mice in the 24th week. **j**, **k** Expression of CREG1 and autophagic and lysosomal proteins in the myocardium of *Creg1*^fl/fl^ and *Creg1*-CKO mice (*n* = 3). *Creg1*^fl/fl^: littermate control, *Creg1*-CKO: *Creg1* cardiac knockout mice, DM: diabetic model, *p < 0.05, **p < 0.01 vs. *Creg1*^fl/fl^; ^##^*p* < 0.01 vs. *Creg1*-CKO; ^&^*p* < 0.05, ^&&^*p* < 0.01 vs. *Creg1*^fl/fl^-DM.

Compared with the control group, the DM group of *Creg1*-CKO and *Creg1*^fl/fl^ mice had decreased diastolic and systolic functions at 24 weeks (*p* < 0.01, Fig. 1a–c). Interestingly, diastolic function (E/A) and systolic function (EF% and FS%) were significantly worse in the DM group of *Creg1*-CKO mice than in the DM group of *Creg1*^fl/fl^ mice at 24 weeks (E/A: 1.20 ± 0.03 vs. 1.41 ± 0.08; *p* < 0.05; EF%: 30.74% ± 1.51% vs. 37.39% ± 1.18%, *p* < 0.01; FS%: 14.37% ± 0.78% vs. 17.85% ± 0.60%, *p* < 0.01; Fig. 1a–c).

### CREG1 deficiency exacerbated myocardial hypertrophy and fibrosis in type 2 diabetic mice

In the absence of pathological stimulation, there was mild myocardial hypertrophy in *Creg1*-CKO mice compared with *Creg1*^fl/fl^ mice; however, no significant difference in fibrosis was observed between *Creg1*-CKO and *Creg1*^fl/fl^ mice (Fig. 1d–h). Interestingly, myocardial hypertrophy and fibrosis occurred in the DM group of *Creg1*-CKO and *Creg1*^fl/fl^ mice at 24 weeks compared with the control group (Fig. 1d–h). Moreover, myocardial hypertrophy and fibrosis in the DM group of *Creg1*-CKO mice were more severe than those in the DM group of *Creg1*^fl/fl^ mice (Fig. 1d–h). Furthermore, the mRNA levels of hypertrophic (*Anp* and *Myh7*) and fibrotic (*Tgfβ*) markers in the DM group of *Creg1*-CKO mice were significantly higher than those in the DM group of *Creg1*^fl/fl^ mice (*p* < 0.05, Fig. 1i).

### CREG1 deficiency exacerbated myocardial autophagy dysfunction in type 2 diabetic mice

The expression of LC3II and P62 in *Creg1*-CKO mice was higher than that in *Creg1*^fl/fl^ mice, while the expression of LAMP2 was decreased in *Creg1*-CKO mice (*p* < 0.05, Fig. 1j, k). The expression levels of beclin 1, cathepsin D and LAMP2 were significantly decreased in the DM group of *Creg1*-CKO and *Creg1*^fl/fl^ mice compared with those in the control group. Conversely, the expression levels of LC3II and P62 were significantly increased in the DM group of *Creg1*-CKO and *Creg1*^fl/fl^ mice (*p* < 0.01, Fig. 1j, k). The expression levels of beclin 1 and cathepsin D were not changed in the DM group of *Creg1*-CKO mice compared with those in the DM group of *Creg1*^fl/fl^ mice; however, the expression levels of LC3II and P62 were significantly increased, whereas the expression of LAMP2 was significantly decreased in the DM group of *Creg1*-CKO mice (*p* < 0.01, Fig. 1j, k).

To further clarify the role of CREG1 deficiency in cardiac autophagy in vivo, the protein levels of LC3II, P62 and LAMP2 in the myocardium of *Creg1*-CKO and *Creg1*^fl/fl^ mice with or without colchicine treatment were examined. Colchicine increased the protein expression of LC3II, P62 and LAMP2 in *Creg1*^fl/fl^ mice and *Creg1*-CKO mice. Compared with that in the *Creg1*^fl/fl^ + colchicine group, the protein expression of LC3II and P62 was significantly increased in the myocardium in the *Creg1*-CKO + colchicine group (Supplementary Fig. 2d, e). These results indicated that CREG1 deficiency exacerbated cardiac autophagy dysfunction.

### CREG1 overexpression improved cardiac function in type 2 diabetic mice

To further clarify the role of CREG1 overexpression in diabetic cardiomyopathy, we used *Creg1*-TG and WT mice to establish DM. We generated *Creg1*-TG mice (Supplementary Fig. 3a–c) and found that the mRNA and protein levels of CREG1 were significantly increased in the myocardium of *Creg1*-TG mice compared with those in the WT mice (*p* < 0.01, Supplementary Fig. 3d–f). Body weight and fasting blood glucose levels were significantly increased in the DM group of *Creg1*-TG and WT mice compared with those in the control group. In addition, body weight and fasting blood glucose levels in the DM group of *Creg1*-TG mice were slightly lower than those in the DM group of WT mice (*p* < 0.05, Supplementary Fig. 3g, h). The HW/TL ratio was significantly higher in the DM group of WT mice than in the control group. The HW/TL ratio in the DM group of *Creg1*-TG mice was lower than that in the DM group of WT mice (*p* < 0.05, Supplementary Fig. 3i).

**Fig. 2:**
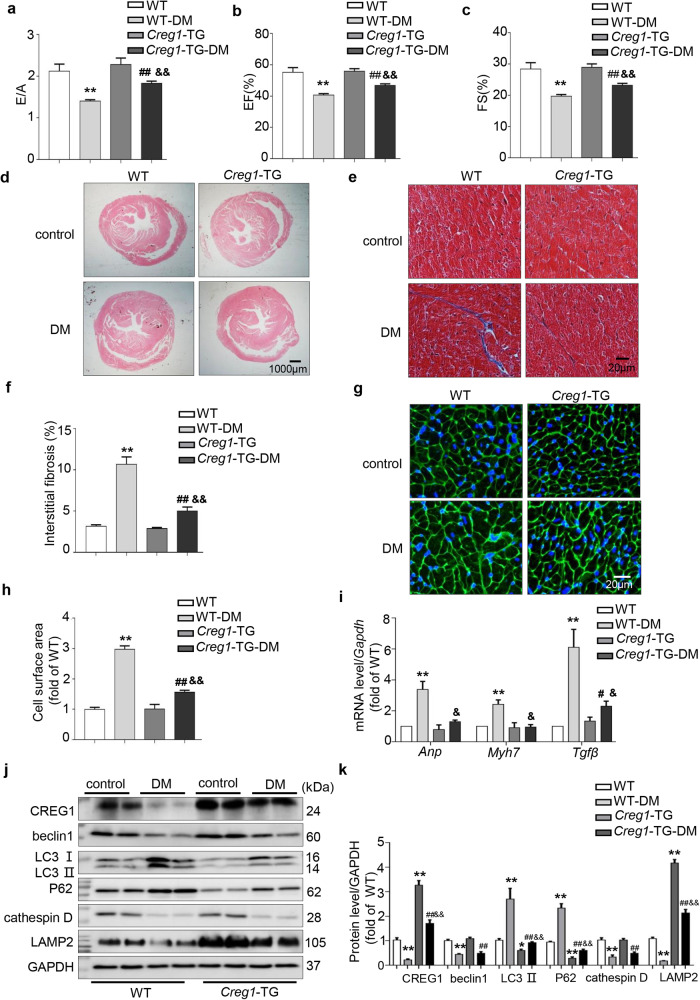
CREG1 overexpression ameliorates cardiac hypertrophy and autophagy dysfunction in type 2 diabetic mice. **a** The E/A ratio in WT and *Creg1*-TG mice after type 2 DM was established (24 weeks, *n* = 7). **b**, **c** EF% and FS% in WT and *Creg1*-TG mice after type 2 DM was established (24 weeks, *n* = 7). **d**–**f** HE staining and Masson’s trichrome staining of control and DM mice in the 24th week (*n* = 3). **g**, **h** WGA staining of the myocardium of control and DM mice in the 24th week (*n* = 4). **i** Effects of CREG1 overexpression on the mRNA levels of hypertrophic (*Anp* and *Myh7*) and fibrotic (*Tgfβ*) marker genes in the myocardium of control and DM mice in the 24th week. **j**, **k** Expression of CREG1, autophagy-related proteins and lysosome-related proteins in the myocardium of WT and *Creg1*-TG mice after DM was established (*n* = 3). WT: littermate control, *Creg1*-TG: *Creg1* transgenic mice, DM: diabetic model. *p < 0.05, **p < 0.01 vs. WT; ^#^*p* < 0.05, ^##^*p* < 0.01 vs. *Creg1*-TG; ^&^*p* < 0.05, ^&&^*p* < 0.01 vs. WT-DM.

Diastolic and systolic function were decreased in the DM group of *Creg1*-TG and WT mice at 24 weeks compared with that in the control group (*p* < 0.01, Fig. 2a–c). Interestingly, diastolic (E/A) and systolic (EF% and FS%) functions were improved in the DM group of *Creg1*-TG mice compared with those in the DM group of WT mice (E/A: 1.83 ± 0.05 vs. 1.41 ± 0.03, respectively; EF%: 46.79% ± 1.06% vs. 40.73% ± 0.84%, respectively; FS%: 23.19% ± 0.62% vs. 19.75% ± 0.49%, respectively; *p* < 0.01, Fig. 2a–c).

### CREG1 overexpression alleviated myocardial hypertrophy and fibrosis in type 2 diabetic mice

At baseline, myocardial hypertrophy and fibrosis were not observed in the *Creg1*-TG and WT groups. However, myocardial hypertrophy and fibrosis occurred in the DM group of *Creg1*-TG and WT mice at 24 weeks (Fig. 2d–h). Interestingly, myocardial hypertrophy and fibrosis were alleviated in the DM group of *Creg1*-TG mice compared with those in the DM group of WT mice (Fig. 2d–h). In addition, the mRNA levels of *Anp*, *Myh7* and *Tgfβ* in the DM group of *Creg1*-TG mice were significantly lower than those in the DM group of WT mice (*p* < 0.05, Fig. 2i).

### CREG1 overexpression improved myocardial autophagy in type 2 diabetic mice

Under physiological conditions, the expression of LC3II and P62 in the myocardium of *Creg1*-TG mice was lower than that in WT mice, whereas the expression of LAMP2 was increased in *Creg1*-TG mice (*p* < 0.05, Fig. 2j, k). Interestingly, the expressions of beclin 1 and cathepsin D in the DM group of *Creg1*-TG mice did not change compared with those in the DM group of WT mice; however, the expression of LC3II and P62 was significantly decreased, whereas that of LAMP2 was significantly increased in the DM group of *Creg1*-TG mice (*p* < 0.01, Fig. 2j, k).

### CREG1 knockdown increased triglyceride and NEFA levels, and CREG1 overexpression inhibited triglyceride and NEFA levels in the myocardium in type 2 diabetic mice

Compared with those in *Creg1*^fl/fl^ mice, triglyceride, NEFA and total cholesterol levels were significantly increased in the DM group of *Creg1*^fl/fl^ mice (*p* < 0.05, Supplementary Fig. 4a–c). Moreover, triglyceride and NEFA levels in the DM group of *Creg1*-CKO mice were higher than those in the DM group of *Creg1*^fl/fl^ mice (*p* < 0.05, Supplementary Fig. 4a, b). However, HDL-C and LDL-C levels did not change in the DM group (*p* > 0.05, Supplementary Fig. 4d, e,i, j). In addition, compared with those in WT mice, triglyceride, NEFA and total cholesterol levels were significantly increased in the DM group of WT mice (*p* < 0.05, Supplementary Fig. 4f–h). Triglyceride and NEFA levels were significantly decreased in the DM group of *Creg1*-TG mice compared to those in the DM group of WT mice (*p* < 0.05, Supplementary Fig. 4f, g).

### CREG1 knockdown exacerbated cardiomyocyte hypertrophy in vitro

**Fig. 3:**
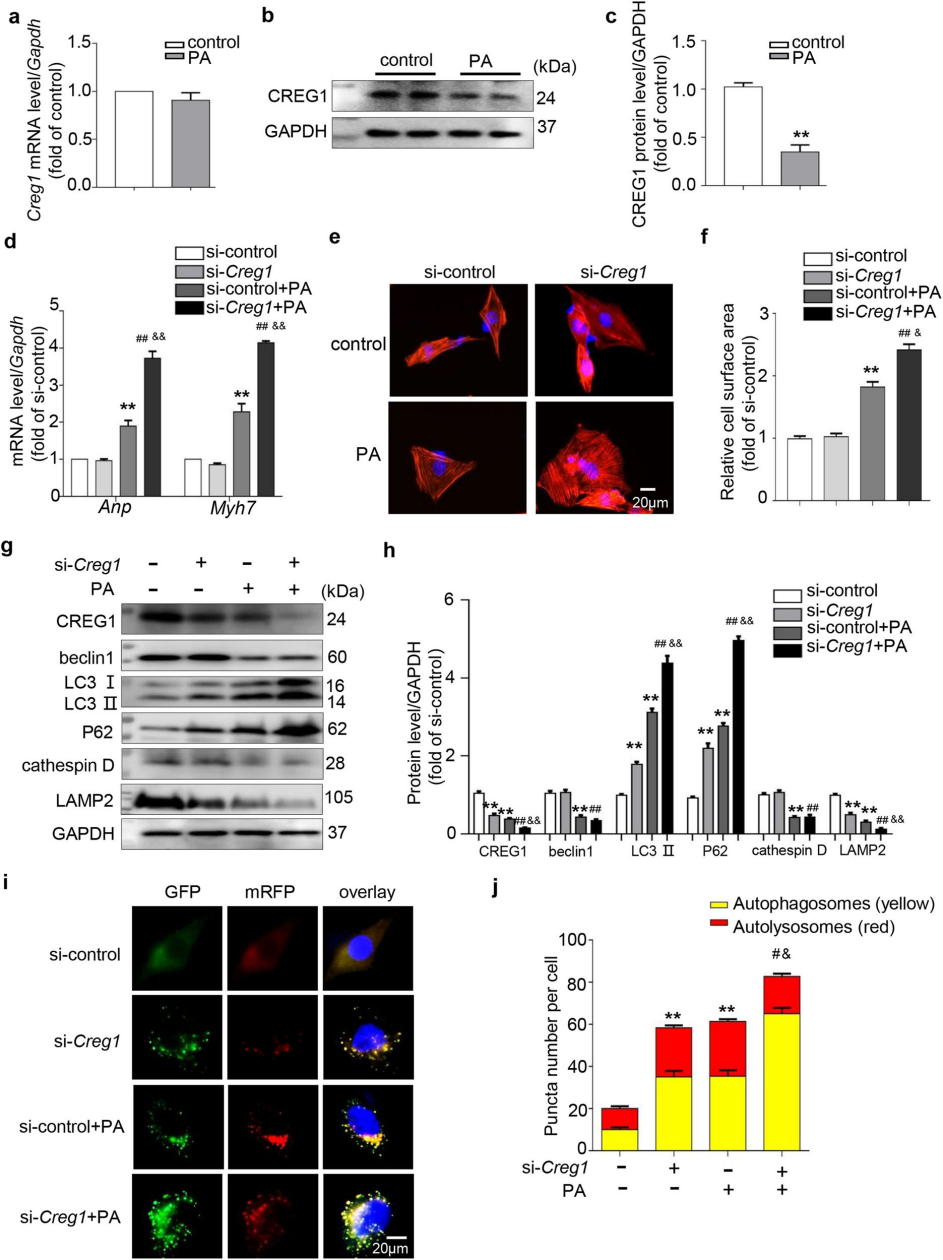
CREG1 deficiency exacerbates palmitate-induced autophagy dysfunction in cardiomyocytes. **a** The mRNA expression of *Creg1* in NMCMs after PA stimulation (*n* = 3). **b**, **c** The protein expression of CREG1 in NMCMs after PA stimulation (*n* = 4). **d** Effects of CREG1 knockdown on the mRNA expression of *Anp* and *Myh7* (*n* = 3). **e**, **f** F-actin staining in NMCMs with CREG1 knockdown and PA stimulation (*n* = 5). **g**, **h** Effects of CREG1 knockdown on the expression of autophagy-related proteins and lysosome-related proteins (*n* = 4). **i**, **j** Effects of CREG1 knockdown on autophagic flux in NMCMs (*n* = 3). NMCMs: neonatal mouse cardiomyocytes, PA: palmitate. **p < 0.01 vs. control or si-control, ^#^*p* < 0.05, ^##^*p* < 0.01 vs. si-*Creg1*; ^&^*p* < 0.05, ^&&^*p* < 0.01 vs. si-control+PA.

Previous studies have reported that lipotoxicity is the most important mechanism in the development of diabetic cardiomyopathy^31–34^. Therefore, we constructed a cell model of lipotoxicity by culturing NMCMs in medium containing PA. PA dramatically reduced CREG1 protein expression in NMCMs (*p* < 0.01, Fig. 3a–c). To determine whether CREG1 knockdown enhanced PA-induced cardiomyocyte hypertrophy, NMCMs were transfected with si-*Creg1* and then stimulated with PA. We first examined the mRNA and protein expression of CREG1 in NMCMs after si-*Creg1* transfection. Compared with that in the si-control group, the mRNA and protein expression of CREG1 was decreased in the si-*Creg1* group (Supplementary Fig. 5a–c). PA increased the mRNA levels of *Anp* and *Myh7* and increased cardiomyocyte size (*p* < 0.01, Fig. 3d–f). Interestingly, CREG1 knockdown exacerbated cardiomyocyte hypertrophy in response to PA stimulation (*p* < 0.05, Fig. 3d–f).

### CREG1 knockdown exacerbated autophagy dysfunction in cardiomyocytes in vitro

Compared with those in the si-control group, an increase in the expression of LC3II and P62 and a decrease in the expression of LAMP2 were observed in the si-*Creg1* group (*p* < 0.01, Fig. 3g, h). In addition, PA increased the expression of LC3II and P62 and decreased the expression of beclin 1, cathepsin D, and LAMP2 (*p* < 0.01, Fig. 3g, h). Interestingly, we observed that the expression levels of LC3II and P62 were increased, whereas that of LAMP2 was decreased in the si-*Creg1* + PA group compared with the si-control+PA group (*p* < 0.01, Fig. 3g, h).

In addition, we examined the formation of autophagosomes and autolysosomes in CREG1-knockdown NMCMs. Normal NMCMs exhibited basal autophagy with few autophagosomes and autolysosomes. Compared with that in the si-control group, the proportion of red puncta (autolysosomes) and yellow puncta (autophagosomes) was significantly increased in the si-*Creg1* group (*p* < 0.01, Fig. 3i, j). After PA stimulation, the proportion of autolysosomes and autophagosomes was also increased (*p* < 0.01, Fig. 3i, j). Furthermore, the proportion of autolysosomes was significantly decreased, whereas the proportion of autophagosomes was increased in the si-*Creg1* + PA group compared with the si-control+PA group (*p* < 0.05, Fig. 3i, j).

Following PA stimulation, the number of acidic lysosomes was significantly reduced in the si-*Creg1* and si-control groups (*p* < 0.01, Supplementary Fig. 5d, e); however, no difference in the number of acidic lysosomes was observed between the si-*Creg1* and si-control groups with or without PA stimulation (*p* > 0.05, Supplementary Fig. 5d, e).

### CREG1 overexpression inhibited cardiomyocyte hypertrophy and improved autophagy in vitro

**Fig. 4:**
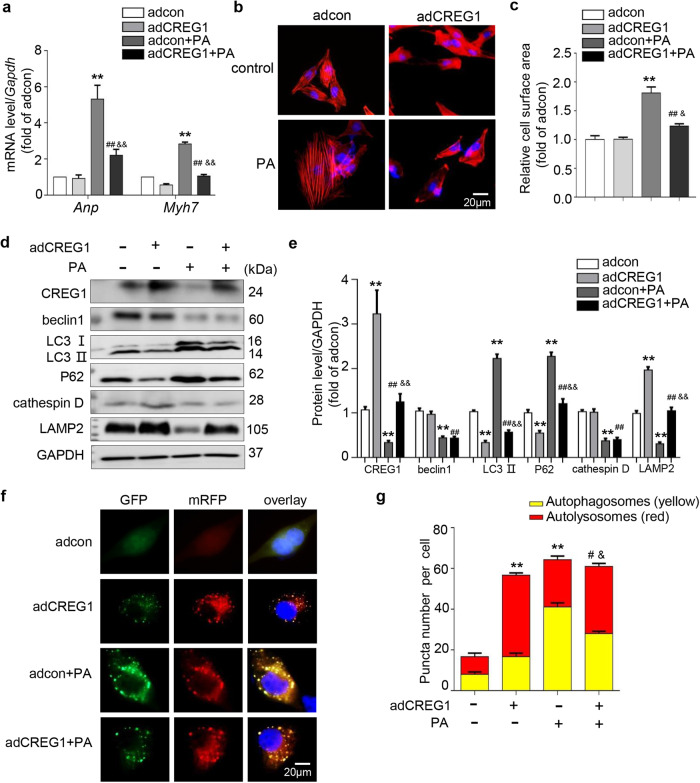
CREG1 overexpression ameliorates palmitate-induced autophagy dysfunction in cardiomyocytes. **a** The mRNA expression of *Anp* and *Myh7* in NMCMs with CREG1 overexpression and PA stimulation (*n* = 3). **b**, **c** F-actin staining in NMCMs with CREG1 overexpression and PA stimulation (*n* = 5). **d**, **e** Effects of CREG1 overexpression on the expression of autophagy-related proteins and lysosomal-related proteins (*n* = 4). **f**, **g** Effects of CREG1 overexpression on autophagosomes and autophagolysosomes in NMCMs (*n* = 3). NMCMs: neonatal mouse cardiomyocytes, PA: palmitate. **p < 0.01 vs. adcon; ^#^*p* < 0.05, ^##^*p* < 0.01 vs. adCREG1; ^&^*p* < 0.05, ^&&^*p* < 0.01 vs. adcon+PA.

To further determine whether CREG1 overexpression improved PA-induced cardiomyocyte hypertrophy and autophagy dysfunction, we infected NMCMs with adCREG1 and stimulated them with PA. CREG1 overexpression inhibited cardiomyocyte hypertrophy by decreasing cardiomyocyte size and inhibiting the mRNA levels of hypertrophic markers (*p* < 0.05, Fig. 4a–c). Compared with that in the adcon group, the expression of LC3II and P62 was decreased, whereas that of LAMP2 was increased in the adCREG1 group (*p* < 0.01, Fig. 4d, e). Moreover, CREG1 overexpression inhibited the expression of LC3II and P62 but increased the expression of LAMP2 in response to PA stimulation (*p* < 0.01, Fig. 4d, e).

In the context of PA stimulation, CREG1 overexpression significantly increased the proportion of autolysosomes but decreased the proportion of autophagosomes (*p* < 0.05, Fig. 4f, g). LysoTracker Red staining indicated that CREG1 overexpression did not affect the number of acidic lysosomes in NMCMs with or without PA stimulation (*p* > 0.05, Supplementary Fig. 5f, g).

### CREG1 knockdown increased the levels of triglycerides and NEFA, and CREG1 overexpression inhibited the levels of triglycerides and NEFA in cardiomyocytes stimulated with PA

Compared with those in the si-control group, the levels of triglyceride, NEFA, total cholesterol, and LDL-C were significantly increased in the si-control+PA group (*p* < 0.01, Supplementary Fig. 6a, b). Moreover, triglyceride and NEFA levels in the si-*Creg1*+PA group were significantly higher than those in the si-control+PA group (*p* < 0.01, Supplementary Fig. 6a). However, HDL-C levels did not change in any group (*p* > 0.05, Supplementary Fig. 6b, d). Triglyceride and NEFA levels were significantly lower in the adCREG1+PA group than in the adcon+PA group (*p* < 0.05, Supplementary Fig. 6c).

### CREG1 overexpression ameliorated cardiomyocyte hypertrophy by activating autophagy

**Fig. 5:**
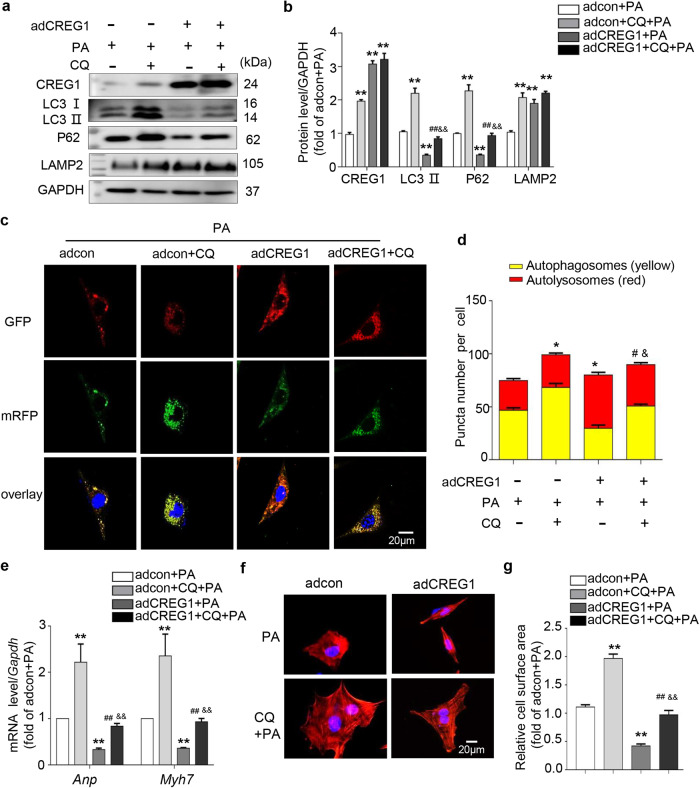
CREG1 overexpression ameliorates cardiomyocyte hypertrophy by activating cardiomyocyte autophagy. **a**, **b** Effects of CREG1 overexpression on the expression of autophagy-related proteins in CQ-induced NMCMs (*n* = 3). **c**, **d** Effects of CREG1 overexpression on autophagosomes and autophagolysosomes in CQ-induced NMCMs (*n* = 3). **e** The mRNA expression of *Anp* and *Myh7* in CREG1-overexpressing NMCMs following CQ stimulation (*n* = 3). **f**, **g** F-actin staining in CREG1-overexpressing NMCMs following CQ stimulation (*n* = 5). NMCMs: neonatal mouse cardiomyocytes, PA: palmitate (400 μM, 24 h), CQ: chloroquine (20 μM, 24 h). *p < 0.05, **p < 0.01 vs. adcon+PA; ^#^*p* < 0.05, ^##^*p* < 0.01 vs. adCREG1+PA; ^&^*p* < 0.05, ^&&^*p* < 0.01 vs. adcon+CQ+PA.

To clarify whether autophagic flux blockade accounted for the accumulation of LC3II and P62, we used different drugs to further inhibit autophagy. CQ (an autophagic degradation inhibitor that changes the lysosomal pH^35^) was incubated with NMCMs for 24 h, and the effect of CQ on NMCM autophagy was examined. CQ increased the expression of LC3II, P62 and LAMP2 under normal and PA conditions (Supplementary Fig. 7a–d, Fig. 5a, b), and the extent of this increase in the PA group was much smaller than that in the normal group. With or without PA stimulation, CREG1 overexpression inhibited the expression of LC3II and P62 induced by CQ (Supplementary Fig. 7c, d, Fig. 5a, b). In addition, we examined the accumulation of autophagosomes and autolysosomes. Compared with that in the control group, CQ treatment increased autophagosomes in NMCMs and inhibited autolysosomes with or without PA stimulation, and these effects were reversed by CREG1 overexpression (Supplementary Fig. 7e, f, Fig. 5c, d). In addition, CQ exacerbated cardiac hypertrophy in response to PA stimulation, which was inhibited by CREG1 overexpression (*p* < 0.01, Fig. 5e–g).

To further examine whether the increase in LC3II and P62 reflects the enhancement of autophagosome formation or accumulation of autophagosomes due to suppressed autolysosome formation, we also used bafilomycin A1 (200 nM), a late autophagic flux inhibitor^36^. Baf A1 increased the expression of LC3II and P62 under normal and PA conditions, and this effect was inhibited by CREG1 overexpression (Supplementary Fig. 8a–d). Bafilomycin A1 increased the number of autophagosomes and inhibited the number of autolysosomes in NMCMs in response to PA stimulation, and these effects were reversed by CREG1 overexpression (Supplementary Fig. 8e, f). These results indicated that the fusion and degradation of autophagosomes and lysosomes were blocked by PA stimulation, and autophagic flux was interrupted in NMCMs stimulated with PA. CREG1 overexpression improved autophagic flux in NMCMs.

### Resveratrol inhibited cardiomyocyte hypertrophy in CREG1-knockdown NMCMs

A previous study demonstrated that resveratrol may be a potential therapeutic agent for diabetic cardiomyopathy through the autophagy signaling pathway^37^; therefore, we administered resveratrol to CREG1-knockdown NMCMs and examined the expression of autophagy-related proteins and the index of cardiomyocyte hypertrophy. The effect of resveratrol on NMCM autophagy was first examined under basal conditions. Resveratrol increased the protein expression of CREG1, LC3II and LAMP2 and decreased the expression of P62, indicating that resveratrol could activate autophagy in NMCMs with or without PA stimulation (Supplementary Fig. 9a–d). In addition, resveratrol inhibited cardiac hypertrophy in the si-control+PA and si-*Creg1* + PA groups (*p* < 0.05, Supplementary Fig. 9e–g).

### CREG1 improved cardiomyocyte autophagy by increasing LAMP2 expression in vitro

**Fig. 6:**
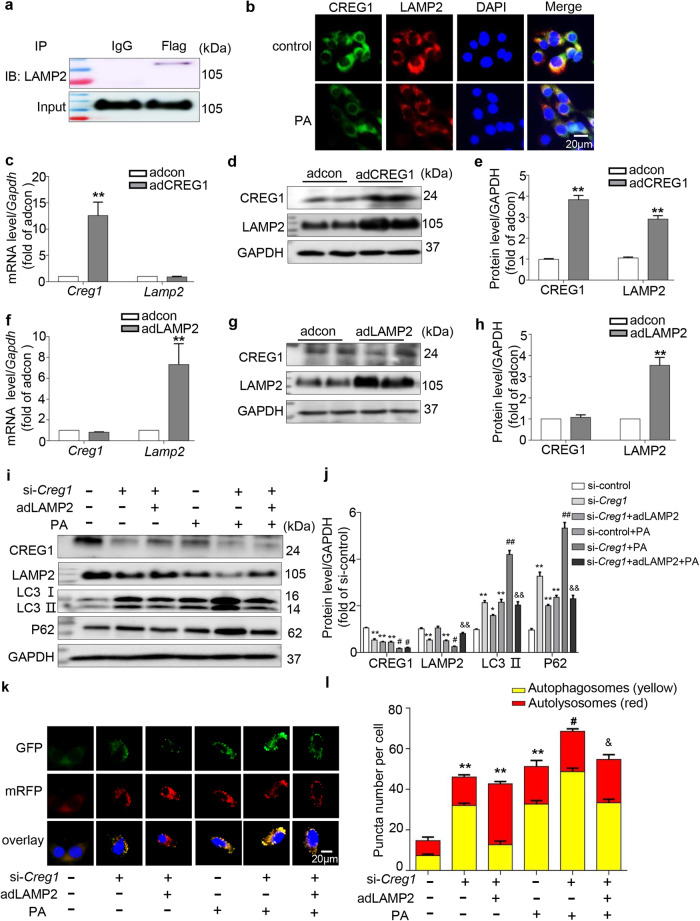
CREG1 deficiency exacerbates autophagy dysfunction in cardiomyocytes by inhibiting LAMP2 protein expression. **a** Immunoprecipitation of CREG1 with LAMP2 in H9C2 cells. **b** Immunofluorescence staining of CREG1 and LAMP2 in NMCMs. **c** Real-time PCR analysis of *Lamp2* mRNA expression in CREG1-overexpressing NMCMs. **d**, **e** Western blotting analysis of LAMP2 protein expression in CREG1-overexpressing NMCMs. **f** Real-time PCR analysis of *Creg1* mRNA expression in LAMP2-overexpressing NMCMs. **g**, **h** Western blotting analysis of CREG1 protein expression in LAMP2-overexpressing NMCMs. **i**, **j** Effects of CREG1 knockdown and LAMP2 overexpression on the expression of autophagy-related proteins and lysosomal-related proteins in NMCMs, as determined by western blotting. **k**, **l** Effects of CREG1 knockdown and LAMP2 overexpression on autophagic flux in NMCMs. NMCMs: neonatal mouse cardiomyocytes, PA: palmitate. *p < 0.05, **p < 0.01 vs. adcon or si-control; ^#^*p* < 0.05, ^##^*p* < 0.01 vs. si-control+PA; ^&^*p* < 0.05, ^&&^*p* < 0.01 vs. si-*Creg1* + PA, *n* = 3.

Previous studies have shown that knocking down CREG1 inhibits the binding of M6P/IGF2R to LAMP2, whereas CREG1 overexpression has the opposite effect^21^. Here, we examined the interaction between CREG1 and LAMP2. There was an interaction between CREG1 and LAMP2 in cardiomyocytes (Fig. 6a). Immunofluorescence staining showed that CREG1 and LAMP2 were colocalized in NMCMs, and this effect was reduced by PA stimulation (Fig. 6b). To clarify the upstream relationship between CREG1 and LAMP2, we infected NMCMs with adCREG1 or adLAMP2 and examined the mRNA and protein expression of CREG1 and LAMP2. Although the mRNA level was unaltered, LAMP2 protein expression was significantly increased in the adCREG1 group (Fig. 6c–e). However, neither the mRNA nor protein levels of CREG1 were affected by LAMP2 overexpression (Fig. 6f–h).

To assess the role of LAMP2 in mediating the effects of CREG1 on the cardiomyocyte autophagy, the effects of LAMP2 overexpression on NMCMs autophagy under basal conditions were first examined. No difference in autophagy occurred between the adLAMP2 and adcon groups (Supplementary Fig. 10a–d). Interestingly, the expression of LC3II and P62 was significantly inhibited in the si-*Creg1*+adLAMP2+PA group compared with that in the si-*Creg1* + PA group (*p* < 0.01, Fig. 6i, j). In addition, LAMP2 overexpression inhibited the accumulation of autophagosomes and increased the accumulation of autolysosomes in CREG1-knockdown NMCMs (*p* < 0.05, Fig. 6k, l).

### CREG1 increased LAMP2 protein expression via a FBXO27-dependent pathway

**Fig. 7:**
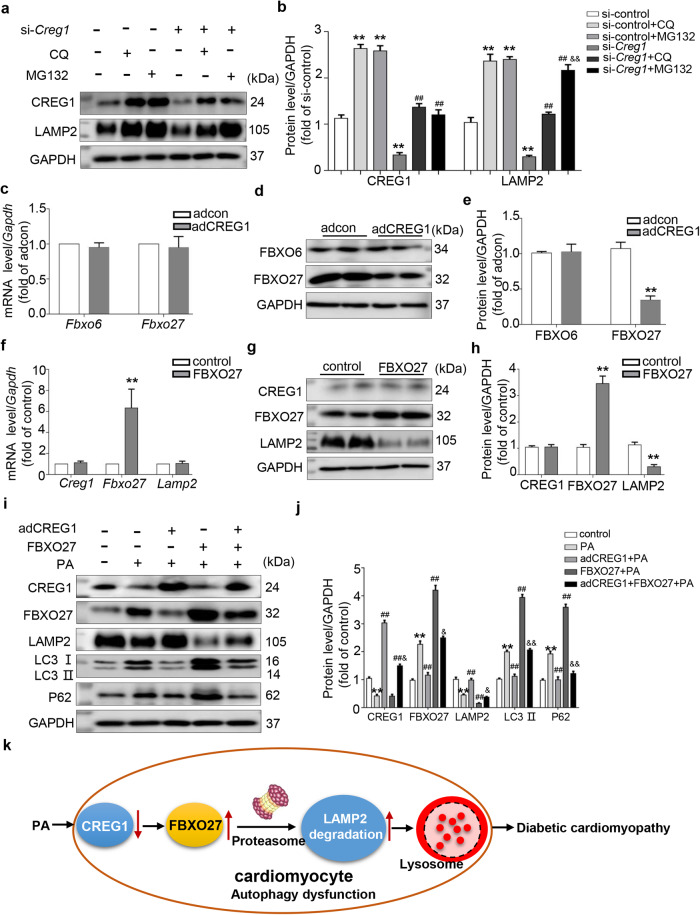
CREG1 inhibits the degradation of LAMP2 in a FBXO27-dependent manner. **a**, **b** Western blotting analysis of LAMP2 or CREG1 protein expression in NMCMs following stimulation with CQ or MG132. **c** Real-time PCR analysis of the mRNA expression of *Fbxo6* and *Fbxo27* in CREG1-overexpressing NMCMs. **d**, **e** Western blotting analysis of the protein expression of FBXO6 and FBXO27 in CREG1-overexpressing NMCMs. **f** Real-time PCR analysis of the mRNA expression of *Creg1* and *Lamp2* in FBXO27-overexpressing NMCMs. **g**, **h** Western blotting analysis of the protein expression of CREG1 and LAMP2 in FBXO27-overexpressing NMCMs. **i**, **j** Effects of CREG1 and FBXO27 overexpression on the expression of autophagy-related proteins in NMCMs, as determined by western blotting. **k** Schematic illustration of the proposed mechanism of CREG1 in diabetic cardiomyopathy. NMCMs: neonatal mouse cardiomyocytes, CQ: chloroquine (20 μM, 24 h), PA: palmitate (400 μM, 24 h). **p < 0.01 vs. si-control or adcon or control; ^##^*p* < 0.01 vs. si-*Creg1* or PA*;* ^*&*^*p* < 0.05, ^*&&*^*p* < 0.01 vs. si-*Creg1* + CQ or FBXO27 + PA, *n* = 3.

To examine the role of CREG1 in the protein degradation of LAMP2, we treated CREG1-knockdown NMCMs with CQ or MG132 and measured LAMP2 expression. Compared with that in the control group, LAMP2 protein expression was decreased in the si-*Creg1* group. LAMP2 protein expression was increased in the CQ group and MG132 group. Interestingly, compared with that in the si-*Creg1* group, LAMP2 protein expression was significantly increased in the si-*Creg1* + CQ group and si-*Creg1* + MG132 group. The extent of the increase in LAMP2 protein expression in the si-*Creg1* + MG132 group was significantly greater than that in the si-*Creg1* + CQ group (*p* < 0.01, Fig. 7a, b). Moreover, compared with that in the control group, LAMP2 protein expression was significantly increased in the PA + CQ group and PA + MG132 group, especially in the PA + MG132 group (*p* < 0.01, Supplementary Fig. 10e, f). These results indicated that LAMP2 might be mainly degraded via the proteasome pathway under pathological conditions.

CREG1 might interact with FBXO6, which is a component of the ubiquitin E3 ligase in 3T3 fibroblast cells (Supplementary Fig. 11a). A previous study revealed that LAMP2 could be degraded by FBXO27 via a proteasome-dependent pathway when lysosomal function was impaired^38^. Therefore, we investigated the interaction between CREG1 and FBXO6 or FBXO27 in cardiomyocytes. Our results demonstrated a lack of any direct interaction between CREG1 and FBXO6 or FBXO27 (Supplementary Fig. 11b, c). We also examined the effect of CREG1 overexpression on the mRNA and protein expression of FBXO6 and FBXO27 in NMCMs. We found that CREG1 did not affect the mRNA and protein expression of FBXO6; however, CREG1 overexpression significantly inhibited FBXO27 protein expression (*p* < 0.01, Fig. 7c–e). In addition, we observed that FBXO27 overexpression did not affect the mRNA and protein expression of CREG1 (*p* > 0.05, Fig. 7f–h) but could inhibit LAMP2 protein expression (*p* < 0.01, Fig. 7g, h). The IP results revealed an interaction between FBXO27 and LAMP2 in cardiomyocytes (Supplementary Fig. 11d). Oddly, we discovered that the mRNA and protein expression of FBXO27 was not affected by LAMP2 overexpression (*p* > 0.05, Supplementary Fig. 11e–g).

To clarify the role of FBXO27 in mediating the effects of CREG1 on cardiomyocyte autophagy, rescue experiments were performed. FBXO27 overexpression significantly inhibited autophagy in NMCMs by increasing the expression of LC3II and P62 and decreasing LAMP2 expression. In addition, the effects of CREG1 on NMCM autophagy were reversed by FBXO27 overexpression (*p* < 0.05, Fig. 7i, j). These results revealed that CREG1 increased LAMP2 protein expression via a FBXO27-dependent pathway (Fig. 7k).

## Discussion

Cardiovascular disease is the leading cause of death worldwide^39^. Diabetic cardiomyopathy is a fatal and chronic complication of diabetes. Autophagy is an important protective mechanism in cells that plays a critical role in the pathogenesis of diabetic cardiomyopathy^40–42^. In our study, the expression of LC3II and P62 was increased, whereas the expression of beclin 1, LAMP2, and cathepsin D was inhibited in the myocardium during diabetic cardiomyopathy and in PA-induced NMCMs. In addition, autophagic flux was inhibited in NMCMs following PA stimulation. These results indicated that autophagy was inhibited during the development of diabetic cardiomyopathy, which was consistent with the findings of previous studies^43–45^. Our results showed that CREG1 expression was decreased in the myocardium during diabetic cardiomyopathy preceding cardiac dysfunction, indicating a negative correlation between the decrease in CREG1 expression and the development of diabetic cardiomyopathy. To determine whether CREG1 plays an important role in diabetic cardiomyopathy, *Creg1*-CKO or *Creg1*-TG mice were used in our study. CREG1 deficiency exacerbated cardiac dysfunction and myocardial fibrosis in diabetic conditions. In contrast, improvements in cardiac function and a decrease in myocardial fibrosis were observed in CREG1-overexpressing diabetic mice.

Autophagy mainly includes the formation of autophagosomes, the fusion of autophagosomes and lysosomes, and the digestion and degradation of metabolites by lysosomes^45^. Autophagy dysfunction in cardiomyocytes in diabetic cardiomyopathy mainly occurs at the stage of autophagy‒lysosome fusion, which is characterized as the reduction and dysfunction of autophagolysosomes^46^. Our results showed that CREG1 deficiency increased the expression of LC3II and P62 and inhibited LAMP2 expression in the myocardium of diabetic mice. CREG1 overexpression inhibited LC3II and P62 and increased LAMP2 expression. However, CREG1 did not influence beclin 1 or cathepsin D expression. The effects of CREG1 on cardiomyocyte autophagy were examined in vitro. In response to PA stimulation, CREG1 deficiency increased LC3II and p62 expression and decreased LAMP2 expression. Conversely, CREG1 overexpression inhibited the expression of LC3II and p62 but increased LAMP2 expression. Autophagic flux is the gold standard to determine whether autophagy is enhanced or inhibited. In this study, CREG1 overexpression in cardiomyocytes increased the formation of autolysosomes and inhibited the formation of autophagosomes, and these effects were reversed by the autophagy inhibitor CQ or bafilomycin A1, whereas CREG1 deficiency increased the formation of autophagosomes and inhibited the formation of autolysosomes. These results revealed that CREG1 improved cardiomyocyte autophagy by promoting the fusion of autophagosomes and lysosomes under diabetic conditions.

LAMP2, which is a lysosomal membrane protein, mediates the fusion of lysosomes and autophagosomes and reflects the function of lysosomes^47^. LAMP2 deficiency inhibits the fusion of lysosomes and autophagosomes in skeletal muscle and the heart, leading to the accumulation of autophagic vacuoles and the suppression of autophagic flux^48^. In our study, LAMP2 overexpression did not affect cardiomyocyte autophagy under basal conditions; however, LAMP2 overexpression could improve autophagy in cardiomyocytes with CREG1 deficiency or PA stimulation. These results were consistent with a previous study^49^. Interestingly, CREG1 increased LAMP2 protein expression without affecting *Lamp2* mRNA expression, indicating that CREG1 could inhibit the protein degradation of LAMP2. FBXO27 is a substrate recognition subunit of the SKP1/CUL1/F-box protein (SCF) ubiquitin ligase complex. FBXO27 can bind and ubiquitinate glycoproteins. In response to lysosomal damage, FBXO27 regulates the autophagic machinery by ubiquitinating lysosomal proteins, including LAMP2. In the present study, CREG1 overexpression increased LAMP2 expression by inhibiting FBXO27 protein expression. In addition, there was an interaction between FBXO27 and LAMP2 in cardiomyocytes. These results indicated that CREG1 increased LAMP2 protein expression via a FBXO27-dependent pathway.

This study has several limitations. First, *Creg1*-TG mice were used in our study. Rescue animal models did not exhibit *Creg1* cardiomyocyte-specific overexpression in vivo. Second, as an extracellular glycoprotein, CREG1 is expected to signal through a surface receptor. However, the CREG1 receptor has not yet been discovered. Finally, when autophagy selectively degrades mitochondria, it is termed mitophagy^50^ and has been reported to be implicated in cardiovascular disorders^51,52^. In our previous study^53^, CREG1 was involved in the regulation of skeletal muscle cell function through mitophagy; however, whether the physiological and pathological role of CREG1 in diabetic cardiomyopathy occurs through mitophagy needs to be investigated in future studies.

Our study revealed that CREG1 could ameliorate the progression of diabetic cardiomyopathy by improving autophagy in cardiomyocytes. These findings provide a new theoretical basis for the prevention and treatment of diabetic cardiomyopathy.

### Supplementary information

Supplementary material

## Data Availability

All data generated or analyzed during this study are included in the published article.

## References

[1] Zheng Y, Ley SH, Hu FB (2018). Global aetiology and epidemiology of type 2 diabetes mellitus and its complications. Nat. Rev. Endocrinol..

[2] Schmidt AM (2019). Diabetes mellitus and cardiovascular disease. Arterioscler. Thromb. Vasc. Biol..

[3] Jia G, Hill MA, Sowers JR (2018). Diabetic cardiomyopathy: an update of mechanisms contributing to this clinical entity. Circ. Res.

[4] Murtaza G (2019). Diabetic cardiomyopathy - a comprehensive updated review. Prog. Cardiovasc. Dis..

[5] Tan Y (2020). Mechanisms of diabetic cardiomyopathy and potential therapeutic strategies: preclinical and clinical evidence. Nat. Rev. Cardiol..

[6] Parim B, Sathibabu Uddandrao VV, Saravanan G (2019). Diabetic cardiomyopathy: molecular mechanisms, detrimental effects of conventional treatment, and beneficial effects of natural therapy. Heart Fail Rev..

[7] Jia G, Whaley-Connell A, Sowers JR (2018). Diabetic cardiomyopathy: a hyperglycaemia- and insulin-resistance-induced heart disease. Diabetologia.

[8] Chen Y (2020). Distinct types of cell death and the implication in diabetic cardiomyopathy. Front. Pharmacol.

[9] Yu L (2022). Activation of silent information regulator 6 signaling attenuates myocardial fibrosis by reducing tgfβ1-smad2/3 signaling in a type 2 diabetic animal model. Cardiol. Discov..

[10] Hu X (2017). Pathophysiological fundamentals of diabetic cardiomyopathy. Compr. Physiol.

[11] Dewanjee S (2021). Autophagy in the diabetic heart: a potential pharmacotherapeutic target in diabetic cardiomyopathy. Ageing Res. Rev.

[12] Kobayashi S, Liang Q (2015). Autophagy and mitophagy in diabetic cardiomyopathy. Biochim. Biophys. Acta..

[13] Varga ZV (2015). Interplay of oxidative, nitrosative/nitrative stress, inflammation, cell death and autophagy in diabetic cardiomyopathy. Biochim. Biophys. Acta..

[14] Parzych KR, Klionsky DJ (2014). An overview of autophagy: morphology, mechanism, and regulation. Antioxid. Redox. Signal..

[15] Dikic I, Elazar Z (2018). Mechanism and medical implications of mammalian autophagy. Nat. Rev. Mol. Cell Biol.

[16] Wu X, Liu Z, Yu XY, Xu S, Luo J (2021). Autophagy and cardiac diseases: therapeutic potential of natural products. Med. Res. Rev..

[17] Ghobrial G, Araujo L, Jinwala F, Li S, Lee LY (2018). The structure and biological function of CREG. Front. Cell Dev. Biol..

[18] Liu Y (2020). DNA hypermethylation: a novel mechanism of CREG gene suppression and atherosclerogenic endothelial dysfunction. Redox. Biol..

[19] Zhang QY (2017). The novel intracellular protein CREG inhibits hepatic steatosis, obesity, and insulin resistance. Hepatology.

[20] Liu J (2021). CREG1 promotes lysosomal biogenesis and function. Autophagy.

[21] Song H (2017). CREG protects from myocardial ischemia/reperfusion injury by regulating myocardial autophagy and apoptosis. Biochim. Biophys. Acta. Mol. Basis Dis..

[22] Liu D (2021). CREG ameliorates the phenotypic switching of cardiac fibroblasts after myocardial infarction via modulation of CDC42. Cell Death Dis..

[23] Zhang J (2018). Transplantation of CREG modified embryonic stem cells improves cardiac function after myocardial infarction in mice. Biochem. Biophys. Res. Commun..

[24] Tai TT (2020). CREG improves cardiac function by regulating cardiomyocytes’ autophagy in diabetic myocardial infarction rats. Eur. Rev. Med. Pharmacol. Sci..

[25] Yan CH (2015). CREG1 ameliorates myocardial fibrosis associated with autophagy activation and Rab7 expression. Biochim. Biophys. Acta..

[26] Gilbert ER, Fu Z, Liu D (2011). Development of a nongenetic mouse model of type 2 diabetes. Exp. Diabetes Res..

[27] Srinivasan K, Viswanad B, Asrat L, Kaul CL, Ramarao P (2005). Combination of high-fat diet-fed and low-dose streptozotocin-treated rat: a model for type 2 diabetes and pharmacological screening. Pharmacol. Res..

[28] Cheng X (2021). Overexpression of kininogen-1 aggravates oxidative stress and mitochondrial dysfunction in DOX-induced cardiotoxicity. Biochem. Biophys. Res. Commun..

[29] Qian P (2020). A novel oral glucagon-like peptide 1 receptor agonist protects against diabetic cardiomyopathy via alleviating cardiac lipotoxicity induced mitochondria dysfunction. Biochem. Pharmacol..

[30] Zhang J (2021). Cardioprotective effect of MLN4924 on ameliorating autophagic flux impairment in myocardial ischemia-reperfusion injury by Sirt1. Redox. Biol..

[31] Yin Z (2019). MiR-30c/PGC-1beta protects against diabetic cardiomyopathy via PPARalpha. Cardiovasc. Diabetol..

[32] Wu MX (2021). Interleukin-33 alleviates diabetic cardiomyopathy through regulation of endoplasmic reticulum stress and autophagy via insulin-like growth factor-binding protein 3. J. Cell Physiol..

[33] Ritchie RH, Abel ED (2020). Basic mechanisms of diabetic heart disease. Circ. Res..

[34] Nakamura M (2019). Glycogen synthase kinase-3alpha promotes fatty acid uptake and lipotoxic cardiomyopathy. Cell Metab..

[35] Moulis M, Vindis C (2017). Methods for measuring autophagy in mice. Cells.

[36] Jung SH (2020). Diclofenac impairs autophagic flux via oxidative stress and lysosomal dysfunction: implications for hepatotoxicity. Redox. Biol.

[37] Kanamori H (2015). Autophagic adaptations in diabetic cardiomyopathy differ between type 1 and type 2 diabetes. Autophagy.

[38] Yoshida Y (2017). Ubiquitination of exposed glycoproteins by SCF(FBXO27) directs damaged lysosomes for autophagy. Proc. Natl. Acad. Sci. USA.

[39] Mei Z, Song H, Tian X, Liu D (2022). Effects of Omega-3 fatty acids on chinese patients with cardiovascular risk factors: a systematic review and meta-analysis. Cardiol. Discov..

[40] Yu W (2017). Sirt3 deficiency exacerbates diabetic cardiac dysfunction: role of Foxo3A-parkin-mediated mitophagy. Biochim. Biophys. Acta Mol. Basis Dis..

[41] Saha S, Panigrahi DP, Patil S, Bhutia SK (2018). Autophagy in health and disease: a comprehensive review. Biomed. Pharmacother..

[42] Kim KH, Lee MS (2014). Autophagy–a key player in cellular and body metabolism. Nat. Rev. Endocrinol.

[43] Yao Q (2018). Curcumin protects against diabetic cardiomyopathy by promoting autophagy and alleviating apoptosis. J. Mol. Cell Cardiol..

[44] Feng Y (2019). LncRNA DCRF regulates cardiomyocyte autophagy by targeting miR-551b-5p in diabetic cardiomyopathy. Theranostics.

[45] D’Arcy MS (2019). Cell death: a review of the major forms of apoptosis, necrosis and autophagy. Cell Biol. Int..

[46] Delbridge LM, Mellor KM, Taylor DJ, Gottlieb RA (2015). Myocardial autophagic energy stress responses–macroautophagy, mitophagy, and glycophagy. Am. J. Physiol. Heart Circ. Physiol..

[47] Alessandrini F, Pezze L, Ciribilli Y (2017). LAMPs: shedding light on cancer biology. Semin. Oncol..

[48] Chi C (2019). LAMP-2B regulates human cardiomyocyte function by mediating autophagosome-lysosome fusion. Proc. Natl. Acad. Sci. USA.

[49] Gu S (2020). Downregulation of LAPTM4B contributes to the impairment of the autophagic flux via unopposed activation of mTORC1 signaling during myocardial ischemia/reperfusion injury. Circ. Res..

[50] Li A (2022). Mitochondrial autophagy: molecular mechanisms and implications for cardiovascular disease. Cell Death Dis..

[51] Wallace KB, Sardao VA, Oliveira PJ (2020). Mitochondrial determinants of doxorubicin-induced cardiomyopathy. Circ. Res..

[52] Wang SH (2021). LncRNA H19 governs mitophagy and restores mitochondrial respiration in the heart through Pink1/Parkin signaling during obesity. Cell Death Dis..

[53] Song H (2021). CREG1 improves the capacity of the skeletal muscle response to exercise endurance via modulation of mitophagy. Autophagy.

