# Supplementary material for: The CREG1-FBXO27-LAMP2 axis alleviates diabetic cardiomyopathy by promoting autophagy in cardiomyocytes
Source: Exp Mol Med. 2023 Sep 1;55(9):2025–38. doi: 10.1038/s12276-023-01081-2 (PMC10545673; doi:10.1038/s12276-023-01081-2)
Supplement: Supplementary file 1 — Supplementary material [file 12276_2023_1081_MOESM1_ESM.pdf]

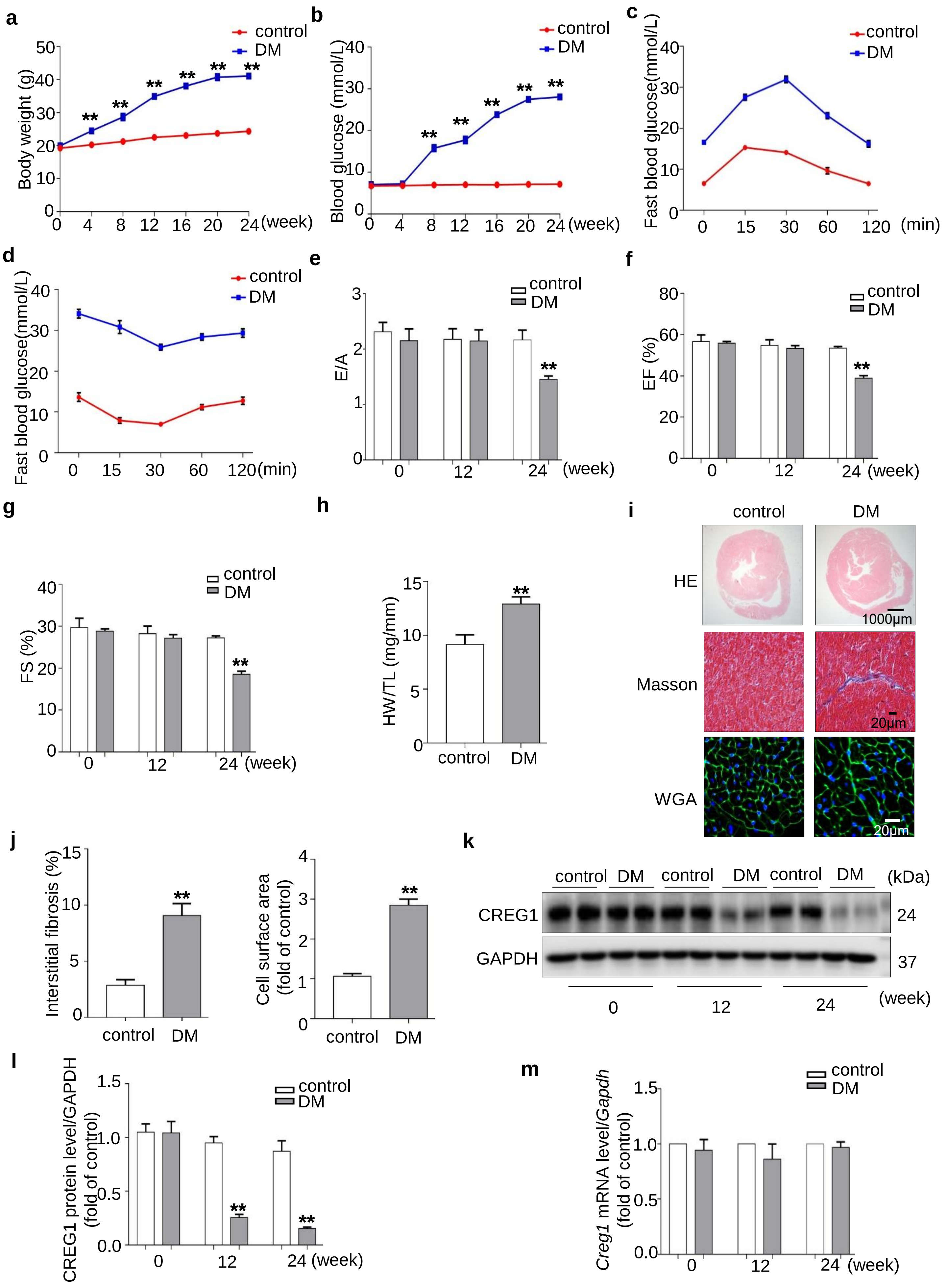

a
control
DM
Body weight (g)
(week)
b
Blood glucose (mmol/L)
c
Fast blood glucose(mmol/L)
(min)
d
e
E/A
f
EF (%)
g
FS (%)
h
HW/TL (mg/mm)
i
HE
Masson
WGA
1000μm
20μm
j
Interstitial fibrosis (%)
Cell surface area (fold of control)
k
CREG1
GAPDH
(kDa)
24
37
l
CREG1 protein level/GAPDH (fold of control)
m
Creg1 mRNA level/Gapdh (fold of control)

Supplementary Fig.1 CREG1 protein expression was decreased in the myocardium of type 2 diabetic mice

a. Body weight of control and DM mice on different time points (0, 4, 8, 12, 16, 20, and 24 week, n = 6). **b**. Levels of fasting blood glucose in control and DM mice on different time points (0, 4, 8, 12, 16, 20, and 24 week, n = 6). **c-d**. Glucose tolerance and insulin tolerance in control and DM mice on 24th week (n = 6). **e**. The E/A ratio in control and DM mice on different time points (0, 12, and 24 week, n = 6). **f-g**. EF% and FS% in control and DM mice on different time points (0, 12, and 24 week, n = 6). **h**. The ratio of heart weight (HW) to tibial length (TL) in control and DM mice on the 24th week (n = 6). **i-j**. HE staining, Masson's trichrome staining, and WGA staining in control and DM mice on the 24th week (n = 3-4). **k-l**. Expression of CREG1 protein in the myocardium of control and DM mice on different time points (0, 12, and 24 week, n = 3). **m**. Expression of *Creg1* mRNA in the myocardium of control and DM mice on different time points (0, 12, and 24 week, n = 3). DM: diabetic model, $^{**}p < 0.01$ vs. control.

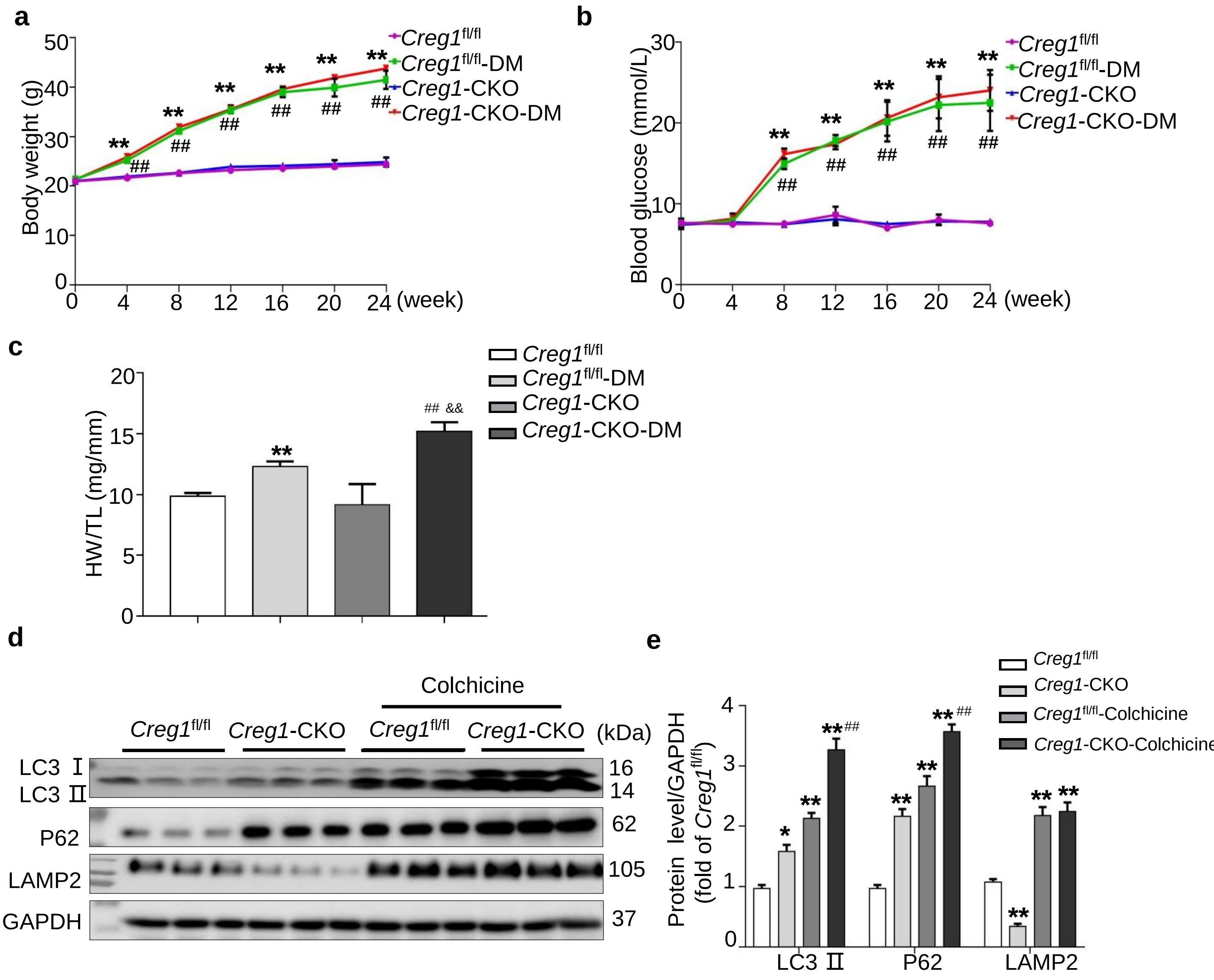

Supplementary Fig.2 CREG1 deficiency aggravated autophagy dysfunction induced by Colchicine

a. Body weight of *Creg1*$^{fl/fl}$ and *Creg1*-CKO mice after establishing the type 2 DM (n = 6). **b**. Fasting blood glucose of *Creg1*$^{fl/fl}$ and *Creg1*-CKO mice after establishing the type 2 DM (n = 6). **c**. The ratio of heart weight (HW) to tibial length (TL) in control and DM mice (n = 6). **d-e**. The protein expressions of autophagy-related protein in the myocardium of *Creg1*$^{fl/fl}$ mice and *Creg1*-CKO mice with or without colchicine treatment (n = 3). *Creg1*-CKO: *Creg1* cardiac-specific knockout mice; *Creg1*$^{fl/fl}$ mice: littermate control mice; Colchicine: intraperitoneally injection (0.4 mg/kg/day for 2 days); $^{*}p < 0.05$, $^{**}p < 0.01$ vs. *Creg1*$^{fl/fl}$ mice; $^{\#\#}p < 0.01$ vs. *Creg1*-CKO or *Creg1*$^{fl/fl}$-Colchicine; $^{\&\&}p < 0.01$ vs. *Creg1*$^{fl/fl}$-DM.

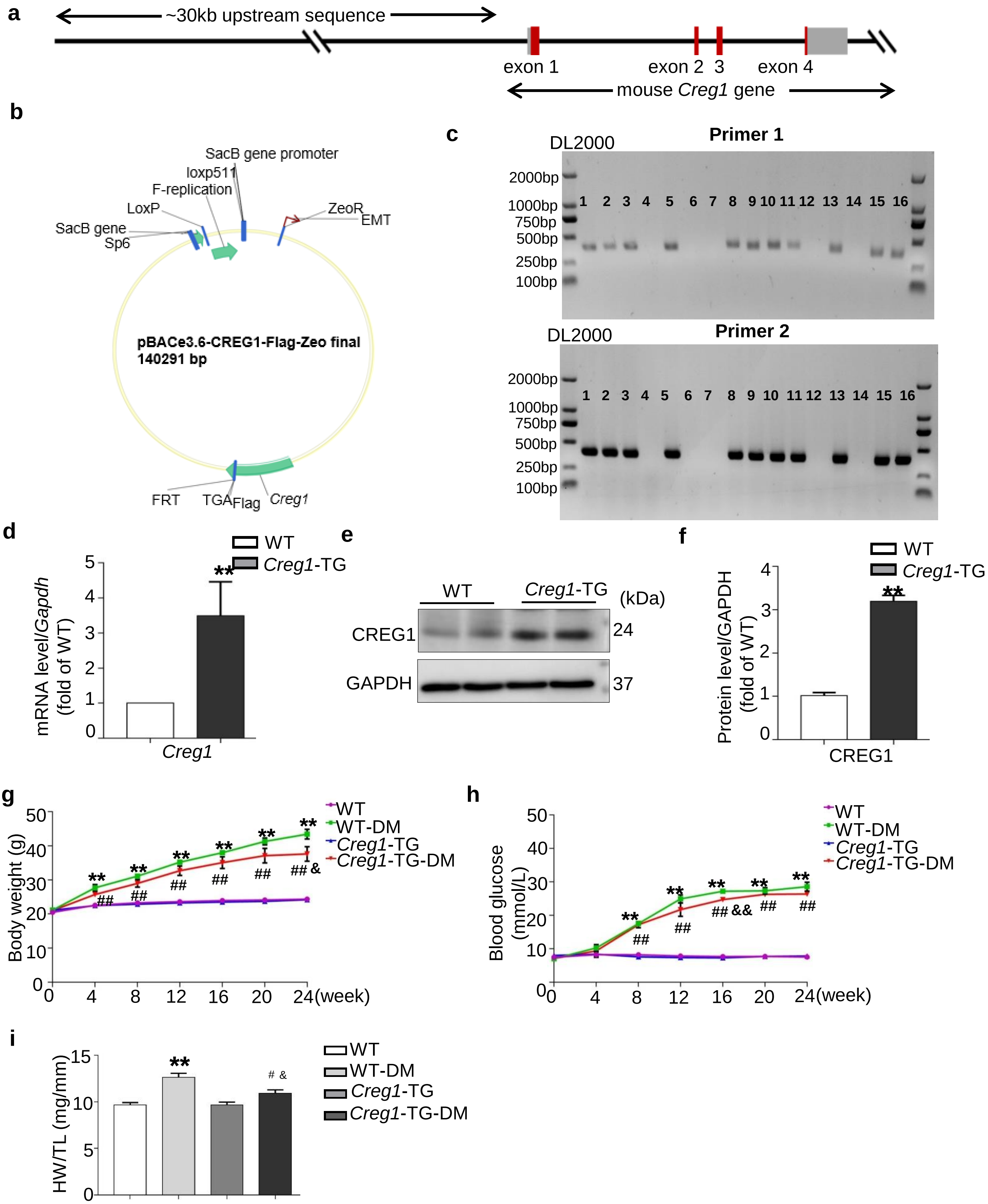

Supplementary Fig.3 The construction of *Creg1* transgenic mice.

a-b. The strategy and map of *Creg1* transgenic mice (*Creg1*-TG). **c**. Genotyping of *Creg1*-TG mice and wild type mice (WT). *Creg1*-TG mice: 1, 2, 3, 5, 8, 9,10,11,13,15 and 16; WT mice: 4, 6, 7, 12 and 14. **d.** *Creg1* mRNA expression in the myocardium of *Creg1*-TG and WT mice (n = 3). **e-f**. CREG1 protein expression in the myocardium of *Creg1*-TG mice and WT mice, n = 3 each group. **g-h**. Body weight and fasting blood glucose of WT and *Creg1*-TG mice after establishing the type 2 DM (n = 7). **i**. The ratio of heart weight (HW) to tibial length (TL) in control and DM mice (n = 7). WT: littermate control, *Creg1*-TG: *Creg* transgenic; DM: diabetic model. **p < 0.01 vs WT; $^{\#}$p < 0.05, $^{\#\#}$p < 0.01 vs. *Creg1*-TG; $^{\&}$p < 0.05, $^{\&\&}$p < 0.01 vs. WT-DM.

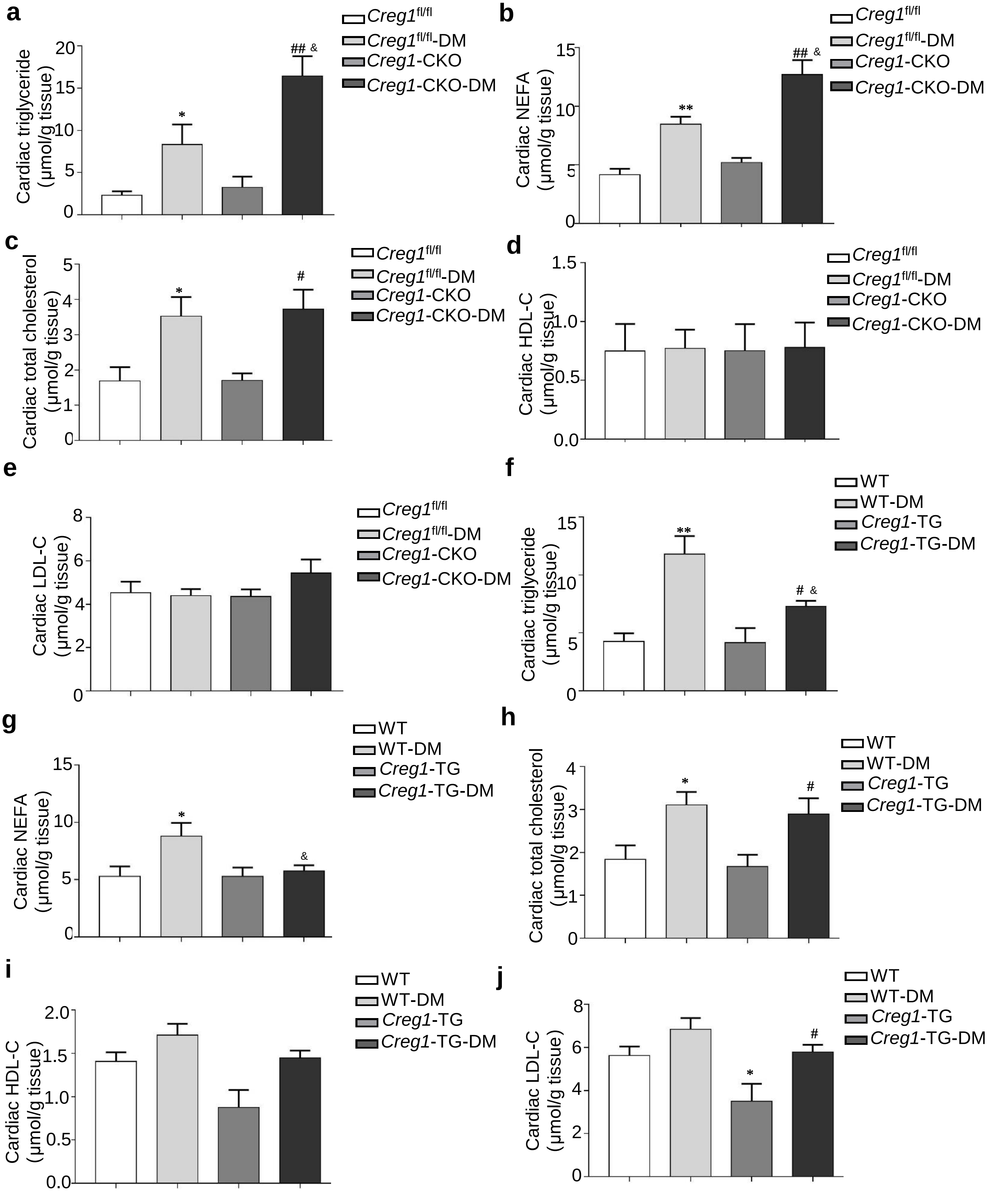

Supplementary Fig.4 Effect of CREG1 knockdown and overexpression on the lipid contents in type 2 diabetic mice

a-e. The content of triglyceride, NEFA, total cholesterol, LDL-C and HDL-C in the myocardium of *Creg1*$^{fl/fl}$ and *Creg1*-CKO mice after establishing the type 2 DM (24 weeks, n = 4). **f-j.** The content of triglyceride, NEFA, total cholesterol, LDL-C and HDL-C in the myocardium of WT and *Creg1*-TG mice after establishing the type 2 DM (24 weeks, n = 4). NEFA: non-esterified fatty acid, LDL-C: low-density lipoprotein-cholesterol, HDL-C: high-density lipoprotein-cholesterol; *Creg1*-CKO: *Creg1* cardiac-specific knockout mice; *Creg1*$^{fl/fl}$ mice: littermate control mice. *$p < 0.05$, **$p < 0.01$ vs. *Creg1*$^{fl/fl}$ mice or WT; $^{\#}p < 0.05$, $^{\#\#}p < 0.01$ vs. *Creg1*-CKO or *Creg1*-TG; $^{\&}p < 0.05$ vs. *Creg1*$^{fl/fl}$-DM or WT-DM.

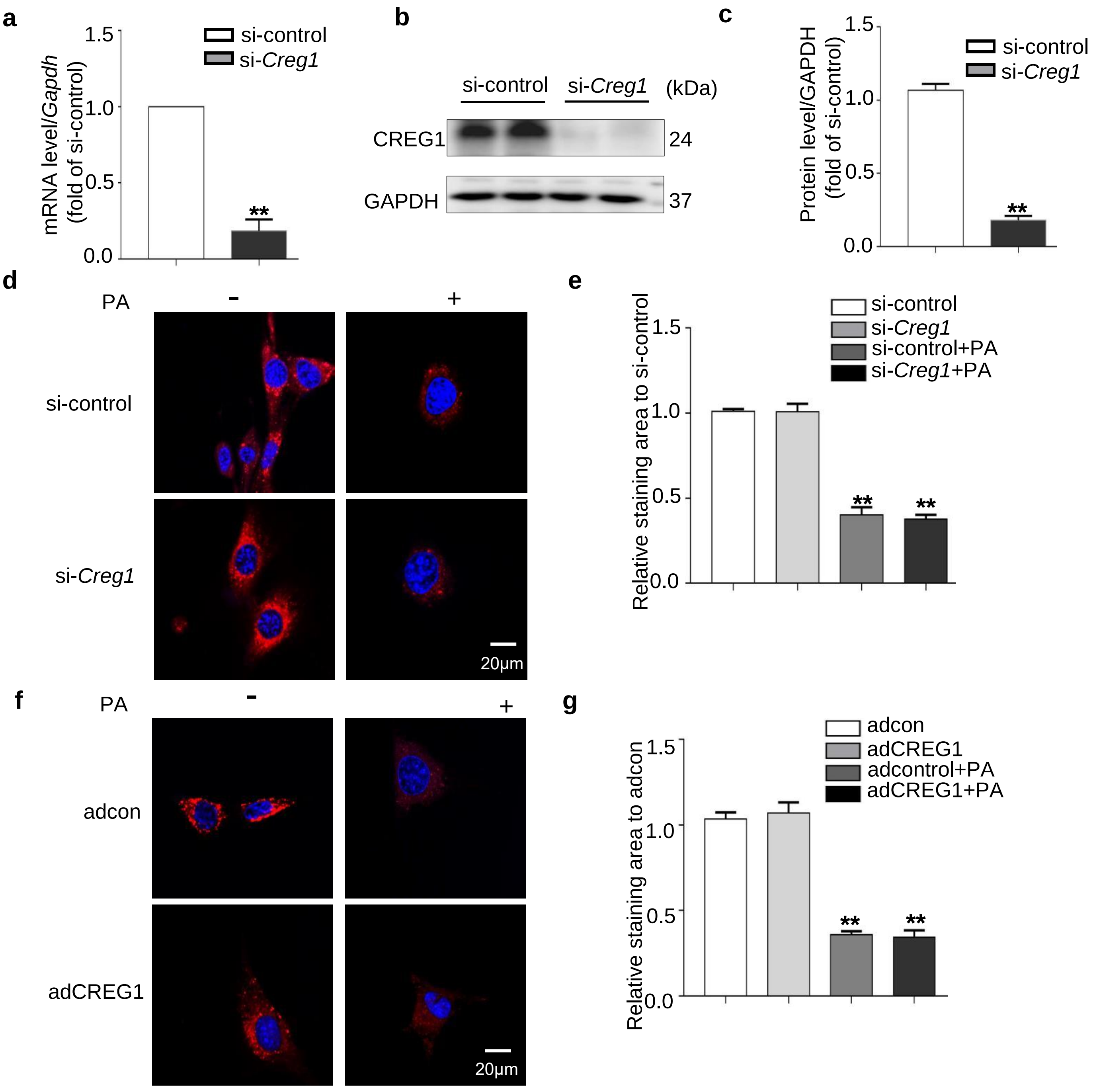

Supplementary Fig.5 CREG1 does not affect lysosomal pH of cardiomyocytes under palmitate stimulation

a. *Creg1* mRNA expression in the NMCMs with si-*Creg1* and si-control transfection (n = 3). **b-c**. CREG1 protein expression in the NMCMs with si-*Creg1* and si-control transfection (n = 3). **d-e**. Lysotracker red staining of NMCMs after CREG1 knockdown and PA stimulation (n = 4). **f-g**. Lysotracker red staining of NMCMs after CREG1 overexpression and PA stimulation (n = 3). NMCMs: neonatal mouse cardiomyocytes; PA: palmitate; $^{**}p < 0.01$ vs. si-control or adcon.

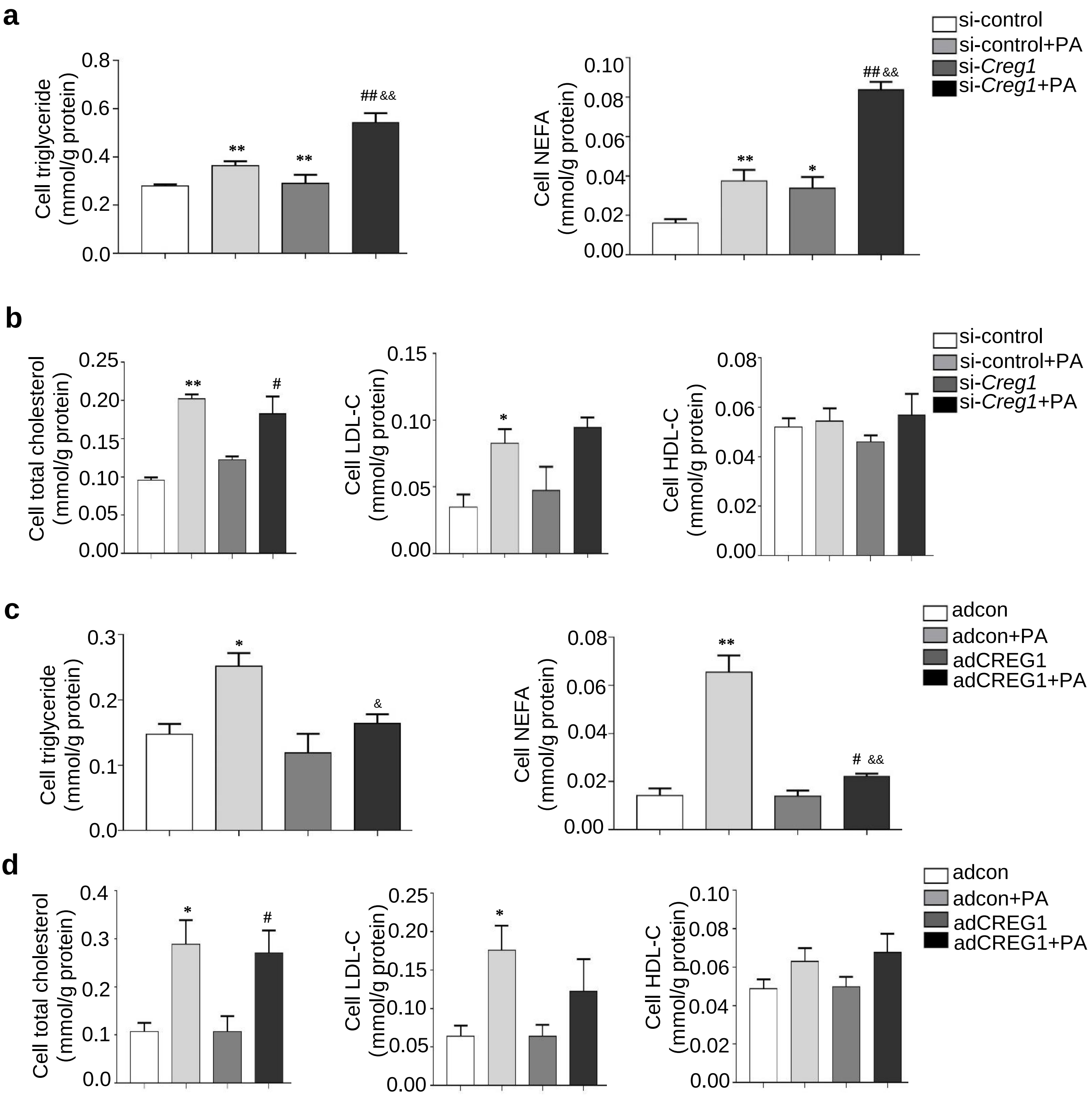

Supplementary Fig.6 Effect of CREG1 knockdown and overexpression on the lipid contents in the cardiomyocytes with PA stimulation

a-b. The content of triglyceride, NEFA, LDL-C, HDL-C and total cholesterol in the cardiomyocytes with CREG1 knockdown and PA stimulation (n = 3). **c-d**. The content of triglyceride, NEFA, LDL-C, HDL-C and total cholesterol in the cardiomyocytes with CREG1 overexpression and PA stimulation (n = 3). PA: palmitate; NEFA: non-esterified fatty acid, LDL-C: low-density lipoprotein-cholesterol, HDL-C: high-density lipoprotein-cholesterol; *$p < 0.05$, **$p < 0.01$ vs. si-control or adcon. $^{\#}p < 0.05$, $^{\#\#}p < 0.01$ vs. si-*Creg1* or adCREG1; $^{\&}p < 0.05$, $^{\&\&}p < 0.01$ vs. si-control+PA or adcon+PA.

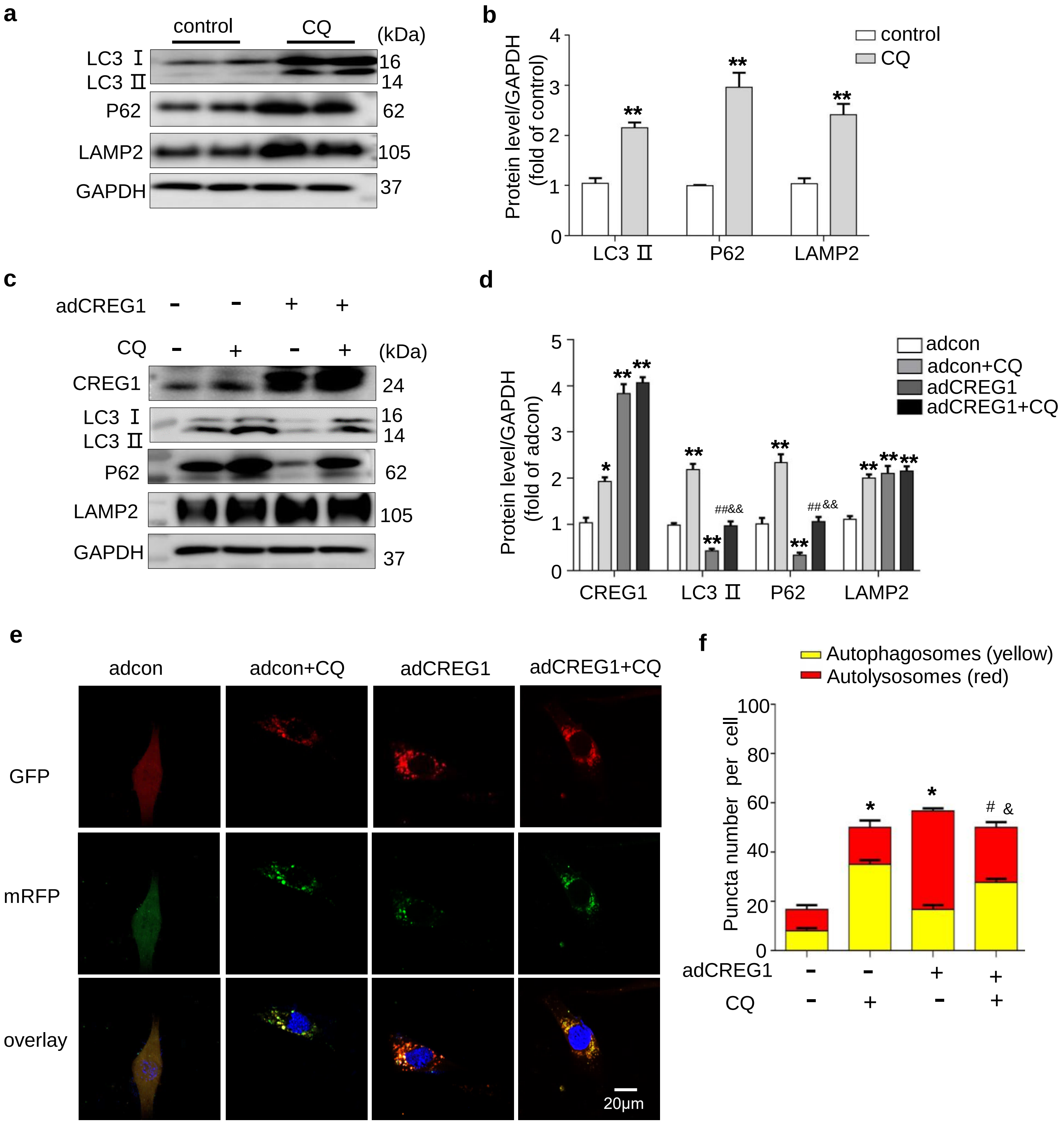

Supplementary Fig.7 Chloroquine inhibited the effect of CREG1 overexpression on the autophagy of cardiomyocytes

a-b. Expression of autophagy-related proteins in NMCMs following CQ stimulation (n = 3). **c-d**. Effects of CREG1 overexpression on the expressions of autophagy-related proteins in CQ-stimulated NMCMs (n = 3). **e-f**. Effects of CREG1 overexpression on the autophagosome and autophagolysosome in CQ-stimulated NMCMs (n = 3). NMCMs: neonatal mouse cardiomyocytes, PA: palmitate (400 μM, 24h), CQ: chloroquine (20 μM, 24h). $^{*}p < 0.05$, $^{**}p < 0.01$ vs. control or adcon; $^{\#}p < 0.05$, $^{\#\#}p < 0.01$ vs. adCREG1; $^{\&}p < 0.05$, $^{\&\&}p < 0.01$ vs. adcon+CQ.

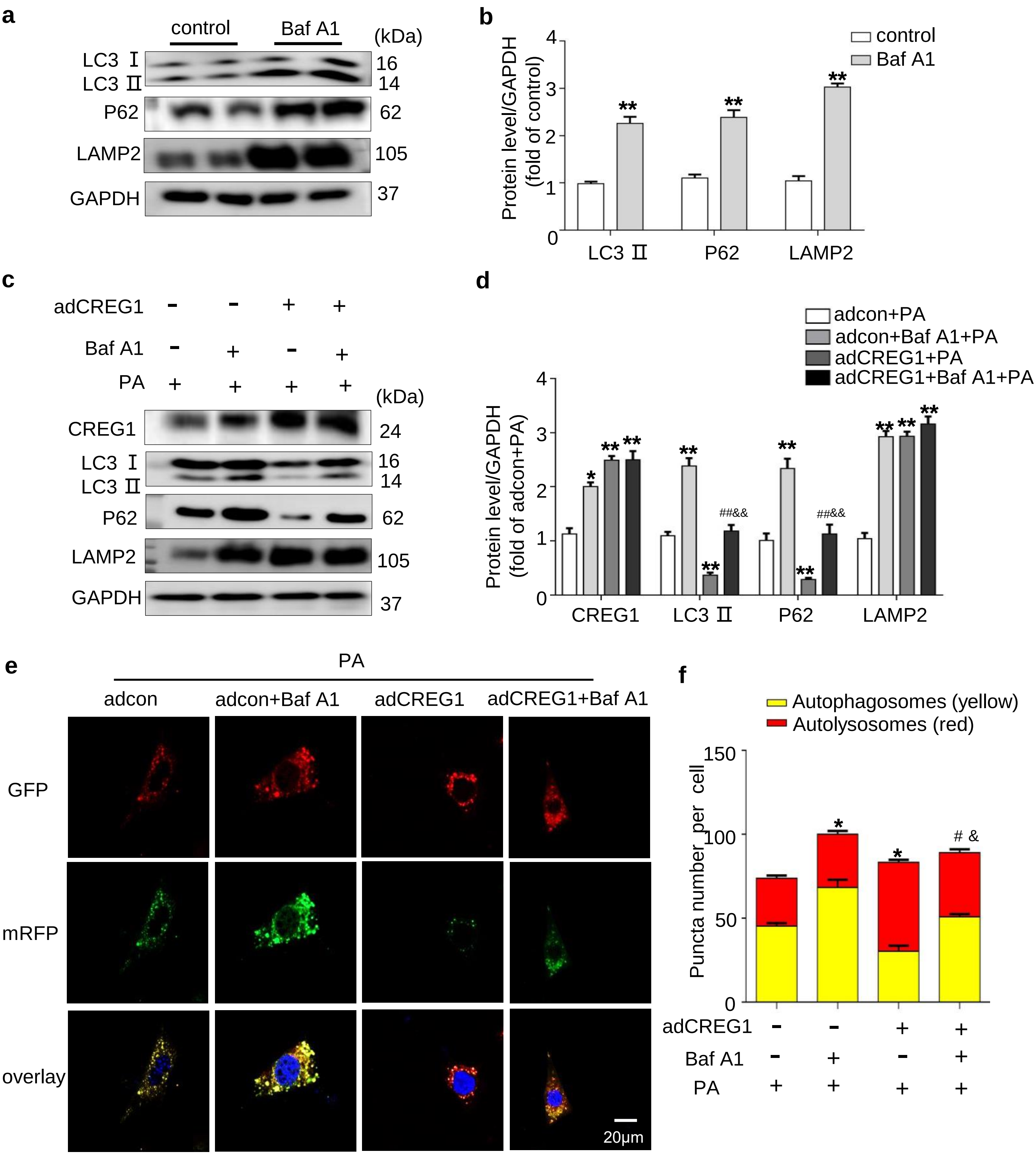

Supplementary Fig.8 Bafilomycin A1 inhibited the effect of CREG1 overexpression on the autophagy of cardiomyocytes under PA stimulation

a-b. Expression of autophagy-related proteins in NMCMs following Baf 1A stimulation (n = 3). **c-d**. Effects of CREG1 overexpression on the expressions of autophagy-related proteins in Baf A1 or PA-stimulated NMCMs (n = 3). **e-f**. Effects of CREG1 overexpression on the autophagosome and autophagolysosome in Baf A1 or PA-stimulated NMCMs (n = 3). NMCMs: neonatal mouse cardiomyocytes, PA: palmitate (400 μM, 24h), Baf A1: Bafilomycin A1 (200 nM, 24h). $^{*}p < 0.05$, $^{**}p < 0.01$ vs. control or adcon+PA; $^{\#}p < 0.05$, $^{\#\#}p < 0.01$ vs. adCREG1+PA; $^{\&}p < 0.05$, $^{\&\&}p < 0.01$ vs. adcon+Baf A1+PA.

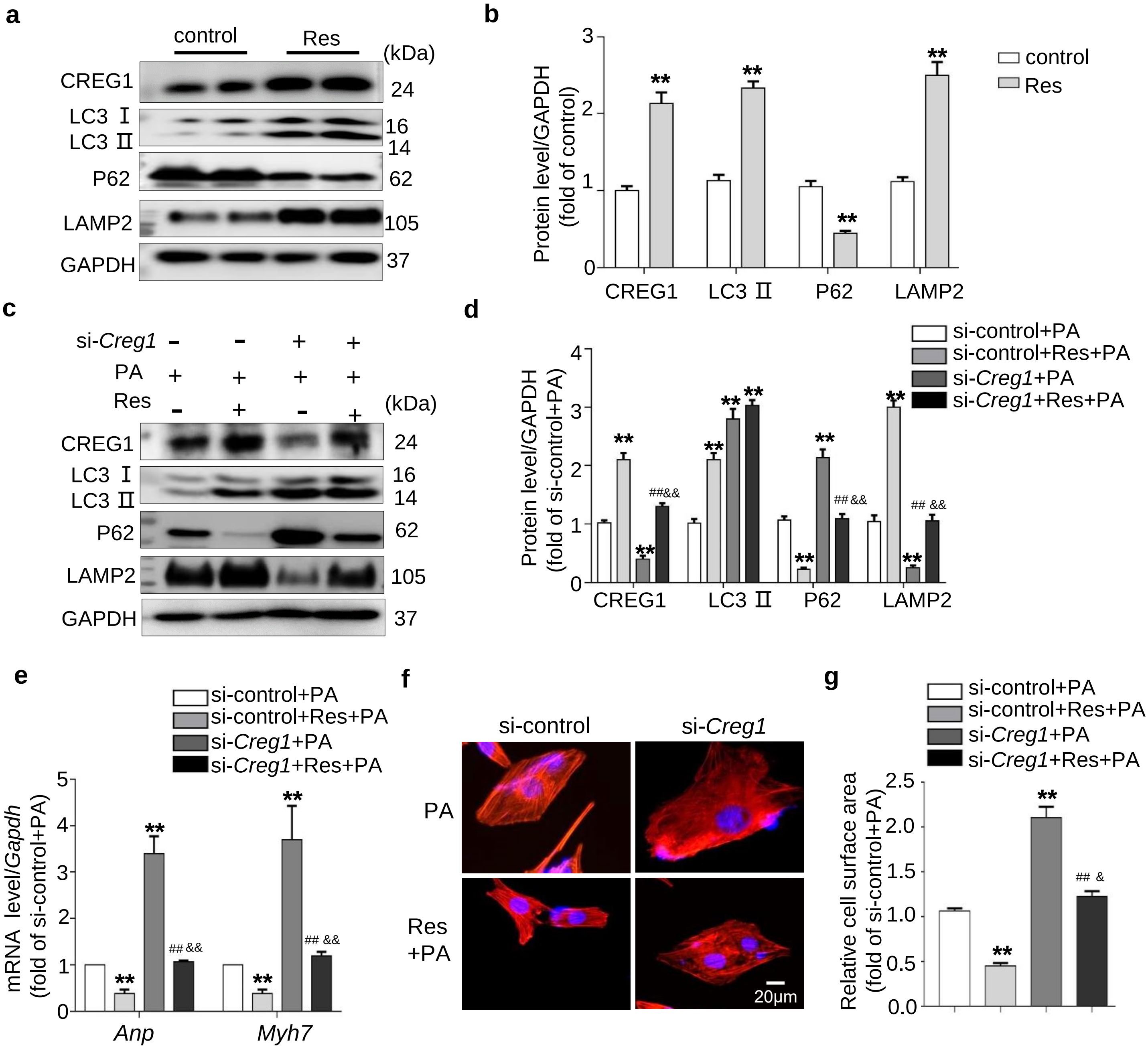

Supplementary Fig.9 Resveratrol reversed the effect of CREG1 knockdown on cardiomyocyte hypertrophy by activating the autophagy of cardiomyocytes

a-b. Expression of CREG1 and autophagy-related proteins in NMCMs following Res stimulation (n = 3). **c-d**. Effects of CREG1 knockdown on the expression of autophagy-related in Res-stimulated NMCMs (n = 3). **e**. Expression of *Anp* and *Myh7* mRNAs in CREG1-knockdown NMCMs following Res stimulation (n = 3). **f-g**. F-actin staining in CREG1-knockdown NMCMs following Res stimulation (n = 5). NMCMs: neonatal mouse cardiomyocytes, PA: palmitate (400 μM, 24h), Res: Resveratrol (25 μM, 24h). $^{**}p < 0.01$ vs. control or si-control+PA; $^{\#\#}p < 0.01$ vs. si-*Creg1*+PA; $^{\&}p < 0.05$, $^{\&\&}p < 0.01$ vs. si-control+Res+PA.

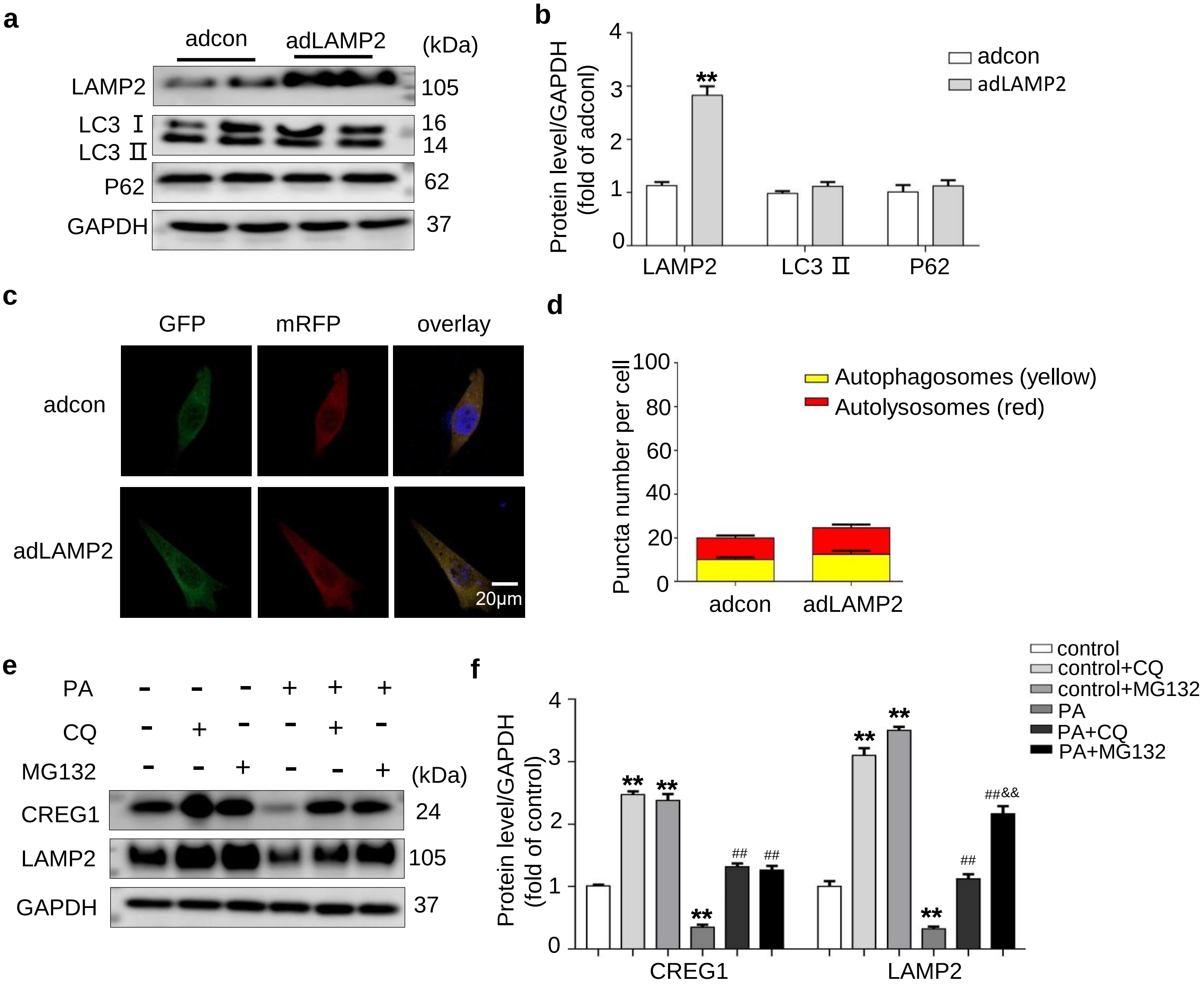

Supplementary Fig.10 Effect of LAMP2 overexpression on the autophagy of cardiomyocytes in the basal condition

a-b. Effects of LAMP2 overexpression on the protein expressions of autophagy-related proteins in NMCMs (n = 3)**. c-d.** Effects of LAMP2 overexpression on the autophagosome and autophagolysosome in NMCMs (n = 3). **e-f**. Western blotting to analyze CREG1 and LAMP2 protein expression in NMCMs following stimulation with CQ or MG132 (n = 3). NMCMs: neonatal mouse cardiomyocytes, PA: palmitate (400 μM, 24h), CQ: chloroquine (20 μM, 24h). $^{**}p < 0.01$ vs. adcon or control; $^{\#\#}p < 0.01$ vs. PA, $^{\&\&}p < 0.01$ vs. PA+CQ.

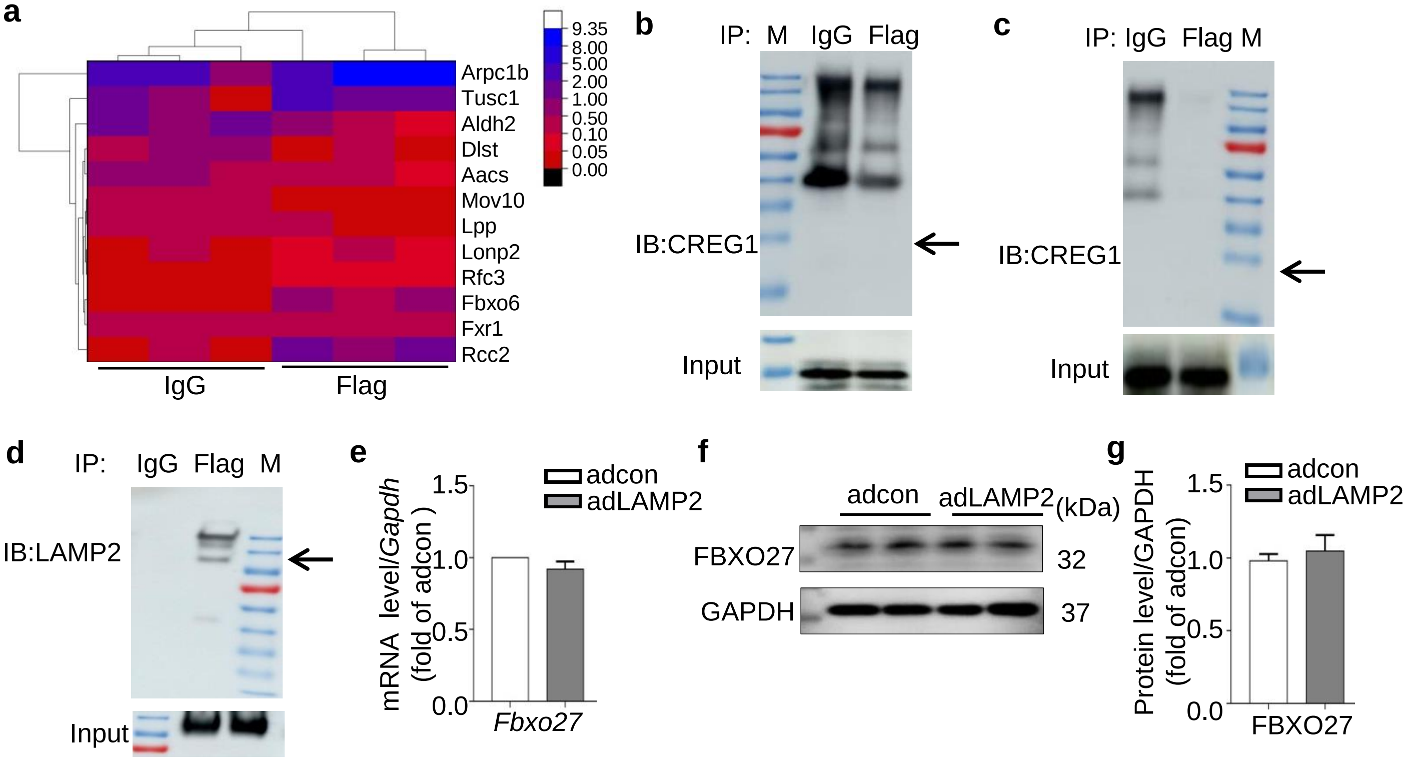

Supplementary Fig.11 Relationship between FBXO27 and CREG or LAMP2 in cardiomyocytes

a. Mass spectrometry in 3T3 fibroblasts with or without CREG1 overexpression. IgG: control group, Flag: CREG1-overexpressed adenovirus (with flag tag). **b**. Immunoprecipitation of FBXO6 with CREG1 in H9C2 cardiomyocytes. IgG: control group, Flag: FBXO6-overexpressed plasmid (with flag tag). **c**. Immunoprecipitation of FBXO27 with CREG1 in H9C2 cardiacmyocytes. IgG: control group, Flag: FBXO27-overexpressed plasmid (with flag tag).**d**. Immunoprecipitation of FBXO27 with LAMP2 in H9C2 cardiomyocytes. IgG: control group, Flag: FBXO27-overexpressing plasmid (with flag tag). **e**. Real-time PCR analysis of the expression of *Fbxo27* mRNA in LAMP2-overexpressing NMCMs (n = 3). **f-g**. Western blotting of FBXO27 protein expression in LAMP2-overexpressing NMCMs (n = 3). NMCMs: neonatal mouse cardiomyocytes.

Supplementary Table 1. Primers for identification of *Creg1* transgenic mice

Primer name	Forward Primer (5'-3')	Reverse Primer (5'-3')
Primer 1	TCTAAAAGACCTAACCGGAA	ACCGGAACGGCACTGGTCAAC
Primer 2	TAATACGACTCACTATAGGGAGA	CAAGCATTTGGCACACAGTGGT

Supplementary Table 2. Primers for real-time PCR

Primer name	Forward Primer (5'-3')	Reverse Primer (5'-3')
Creg1	CTTCGCGGACATCATCTCAAT	GTCAGCGTAGCCTCTGGATTT
Lamp2	TGCCAATTAGGTAAGCAATCACT	TGGCTCAGCTTTCAACATTTCC
Fbxo6	TCCCTATGGAAGCGCAAGAGT	CTCCGTTGGAGTCTATCCGC
Fbxo27	TGAGACCGCTAGGACGCAA	AGTCAGAGACAGCAATCTCCA
Anp	ACCTGCTAGACCACCTGGAG	CCTTGGCTGTTATCTTCGGTACCGG
Myh7	CCGAGTCCCAGGTCAACAA	CTTCACGGGCACCCTTGGA
Tgfβ	GAGCCCGAAGCGGACTACTA	TGGTTTTCTCATAGATGGCGTTG
Gapdh	AGGTCGGTGTGAACGGATTTG	GGGGTCGTTGATGGCAACA
